# *In vitro* and *in vivo* antifungal activity of *Cuminum cyminum* essential oil against *Aspergillus aculeatus* causing bunch rot of postharvest grapes

**DOI:** 10.1371/journal.pone.0242862

**Published:** 2020-11-24

**Authors:** Chutima Tanapichatsakul, Sarunpron Khruengsai, Patcharee Pripdeevech

**Affiliations:** 1 School of Science, Mae Fah Luang University, Muang, Chiang Rai, Thailand; 2 Center of Chemical Innovation for Sustainability (CIS), Mae Fah Luang University, Muang, Chiang Rai, Thailand; Institute for Biological Research "S. Stanković", University of Belgrade, SERBIA

## Abstract

Bunch rot in grapes is an aggressive disease and needs to be controlled during the postharvest period. We investigate the antifungal potential of *Zanthoxylum bungeanum* Maxim., *Zanthoxylum rhetsa*, *Cuminum cyminum*, *Coriandrum sativum*, and *Zingiber montanum* (J. Koenig) Link ex A. Dietr. essential oils against *Aspergillus aculeatus* that cause bunch rot disease on postharvest grapes. *C*. *cyminum* essential oil exhibited stronger significantly inhibition percentage of 95.08% than other treatments in *in vitro* assay. Cumin aldehyde (33.94%) and α-terpinen-7-al (32.20%) were identified as major volatile compounds in *C*. *cyminum* oil. Antifungal potential of *C*. *cyminum* oil was then tested in conidia germination and *in vitro* tests compared to cumin aldehyde and α-terpinen-7-al. Their EC_50_ values against the conidial germination were also estimated. Significant reduction of conidia germination was also detected in *C*. *cyminum* essential oil and cumin aldehyde at a concentration of 1,000 and 100 μg/mL, respectively. EC_50_ values of the *C*. *cyminum* essential oil, cumin aldehyde, and α-terpinen-7-al were 67.28 μg/mL, 9.31 μg/mL, and 13.23 μg/mL, respectively. *In vivo* assay, the decrease of the disease severity (0.69%) and incidence (1.48%) percentage of *A*. *aculeatus* on grape berries treated at 1,000 μg/mL of *C*. *cyminum* essential oil was significantly greater than that obtained from other treatments after 10 days incubation. In addition, grape berries treated with *C*. *cyminum* essential oil decreased weight loss and retained fruit firmness. The changing of total soluble solids, total phenolic content, and antioxidant activity are also delayed in treated fruits. Therefore, essential oil of *C*. *cyminum* may be applied as a biological antifungal agent to control *A*. *aculeatus* in postharvest grapes without any negative effects on its quality.

## 1. Introduction

Grape (*Vitis vinifera* L.) is one of the world’s most abundant fruit crops with various varieties [1]. FAO (2019) reported that grapes are produced at least 50 million metric tons in 2018. It can be cultivated all over the world but mostly in China, which accounts for 16.8% of global production, followed by Italy and the United States at 10.9% and 9.6%, respectively [2]. Wide consumption of grapes and its products, such as wine, juice, jam and raisin showing its importance in worldwide economics. They are also generally known as an essential source of natural antioxidants, including flavonoids, phenolic acids, stilbenes, and lignans [3]. These compounds possess protective effects against many human diseases such as cancer and cardiovascular diseases [4]. In Thailand, grapes can be cultivated in many regions of the country. The Central region is the major production area of table grapes, while the Northeastern and Northern regions produce table and wine grapes. Beauty Seedless is the main grape variety cultivated in the Northern region due to its good growth, production, and quality, followed by Ruby Seedless and Early Muscat [5]. It is considered that grape production in Thailand has been successful. However, an increase in domestic consumption has made it necessary to import grapes from several countries at a value of one hundred million baht per year [6].

Grape quality depends on various factors such as cultivar type, climate, harvest and postharvest periods which affect their firmness, taste, flavor, and defect presence [7, 8]. Quality degradation of grapes is mainly caused by infections from phytopathogens during cultivation and postharvest periods [9]. The major pathogen type that causes significant bunch rot disease in grapes during the postharvest period is the fungal pathogen *Aspergillus aculeatus* [10, 11]. Infection by this pathogen contributes to a significant loss in quality and quantity during the transportation and storage process. Estimation of worldwide production loss is approximately 10 to 40% of total grape production in the world [12, 13]. Sulfur dioxide is typically used to control bunch rot disease in grapes during cultivation and postharvest period [14, 15]. Recently, its use in organic food has been made illegal due to environmental and health hazards, including residual toxicity, carcinogenicity, ecological pollution, and hormonal imbalance [15]. The development of safe and ecofriendly sustainable agriculture has been increased to control postharvest diseases with less impact on human, animal, and environment [16, 17]. Therefore, readily accessible alternative treatments other than sulfur dioxide are needed for controlling bunch rot disease in fresh grapes during postharvest storage, transportation, and market periods.

Over the years, various biological agents have been applied extensively to control diseases from pests and phytopathogens, which is known as biocontrol [18]. Essential oils are considered as biocontrol agents inhibiting phytopathogen growth on fruit surfaces by *in vitro* and *in vivo* assay during the storage period [19]. The use of essential oils controlling postharvest phytopathogens is relatively inexpensive and may not require a sophisticated system. It can be performed by local farmers to extend the shelf lives of their agricultural products and is considered as an environmentally- and user-friendly technique. Although essential oils are advantageous as antimicrobial agent in controlling postharvest diseases, only a few studies have been reported on the control and inhibition of bunch rot disease in fresh grapes caused by *A*. *aculeatus* [10].

*Cuminum cyminum* L., a member of Apiaceae family, is widely known as an aromatic plant. Fruits of this plant is known as cumin seeds containing high amount of essential oil. Cumin seeds have been used therapeutic purposes in traditional medicine [20]. The essential oil of cumin seed is used as natural antioxidants, flavoring the foods and in the treatment of toothache, diarrhea, epilepsy, dyspepsia, and jaundice [20]. Cumin essential oil has been reported to possess antimicrobial activity against various human pathogens such as *Staphylococcus epidermidis*, *Staphylococcus* aureus, *Staphylococcus haemolyticus*, *Propionibacterium acnes*, *Corynebacterium diphtheriae*, and *Candida albicans* [20]. Moreover, this essential oil also exerted antifungal activity against various phytopathogens in postharvest fruits such as *Aspergillus flavus*, *Penicillium italicum*, *Penicillium expansum*, *Penicillium commune*, *Rhizopus stolonifera*, *Rhizopus lyococcus*, and *Botrytis cinerea* [21].

However, this study aimed to investigate inhibitory effect of essential oils produced by *C*. *cyminum* against the growth of *A*. *aculeatus* and its infection on fresh grape berries compared to those essential oils obtained from other plants; *Zanthoxylum bungeanum* Maxim., *Zanthoxylum rhetsa*, *Cuminum cyminum*, *Coriandrum sativum*, and *Zingiber montanum* (J. Koenig) Link ex A. Dietr. These essential oils from selected plants are known as Ayurvedic herbs, which is endemic to tropical Asia and have been used for centuries as herbal medicine to treat various symptoms such as astringent, asthma, bronchitis, diarrhea, and stomach aches. Previous reports have demonstrated that essential oils from these plants showing antimicrobial activities. Thus, the aim of the present study is to investigate the inhibitory effect of these essential oils against *A*. *aculeatus in vitro* and *in vivo* assay. The volatile compounds of plant species were also identified.

## 2. Materials and methods

### 2.1 Plant materials

Healthy *Z*. *bungeanum* Maxim., *Z*. *rhetsa*, *C*. *cyminum*, *C*. *sativum*, and *Z*. *montanum* (J. Koenig) Link ex A. Dietr. were collected from Chiang Rai province, Thailand in May 2019. Age of *Z*. *rhetsa* and *Z*. *montanum* (J. Koenig) Link ex A. Dietr. is approximately seven and two years, respectively. Other plants aged approximately five months. They were washed and further dried at room temperature (27°C) for five days. Each plant species was deposited in the botanical garden of Mae Fah Luang University having a voucher specimen of accession number 10044, 10045, 10046, 10047, and 10048 for *Z*. *bungeanum* Maxim., *Z*. *rhetsa*, *C*. *cyminum*, *C*. *sativum*, and *Z*. *montanum* (J. Koenig) Link ex A. Dietr., respectively.

### 2.2 Extraction of essential oils

Each dried sample was subjected to hydrodistillation for 3 h according to procedures reported by Insawang et al. [22]. Anhydrous Na_2_SO_4_ was added in the obtained essential oils to remove water prior placing pure essential oils in a sealed vial.

### 2.3 Isolation and identification of phytopathogen *A*. *aculeatus*

Isolation of fungal pathogen was performed using a modified method [23]. Infected berries of Beauty Seedless grapes (*Vitis vinifera*) were collected from DoiFang Vineyard Farm (19°51'59.8"N 99°09'25.9"E), Fang district, Chiang Mai province, Thailand in July 2019. The collection site access has been approved by Ms Sawittree Kaewprasert, the owner of DoiFang Vineyard Farm. They were washed with sterile water before placing the fungal spores on Petri plates containing potato dextrose agar (PDA) mixed with chloramphenicol (30 μg/mL) to prevent bacterial growth. The plates were incubated at room temperature for seven days to complete fungal growth. The obtained fungus was further purified using the hyphal tips technique on PDA medium. It was identified based on micro- and macro-morphological characteristics using their cultures and spores according by Adesegun et al. [23]. In addition, the species level of isolated fungus was identified using molecular technique. The genomic DNA of isolated fungus was also extracted using a method of Tanapichatsakul et al. [24]. obtained sequences were compared to all sequences deposited in the National Center for Biotechnology Information database.

### 2.4 *In vitro* test of essential oils on the mycelial growth of *A*. *aculeatus*

The antagonistic activity of essential oils obtained from different plant species against *A*. *aculeatus* which caused bunch rot disease in Beauty Seedless grapes was evaluated using the contact phase method according to Aminifard & Mohammadi [25] and Pedrotti et al. [26]. with some modifications. The contact phase method was carried out using various concentrations of essential oils (200, 400, 600, 800, and 1,000 μg/mL) diluted in 5% v/v Tween 20. PDA (9.0 mL) was placed into 9 cm diameter Petri dish plates. A 100 μL sample of each essential oil solution was placed at the center of a Petri plate. After five minutes, an agar plug (5 mm) of pathogen *A*. *aculeatus* from seven days precultures was placed at the center of the PDA plate. All plates were sealed with Parafilm and further incubated at room temperature for seven days. Mycelia growth was determined after seven days. The mycelial diameter of *A*. *aculeatus* pathogen treated with essential oil in the PDA medium and the control group (no essential oil treatment) was measured daily with a digital Vernier caliper (MITUTOYO-ABS Digimatic Caliper CD-AX) in a mm unit. Plates containing 9.0 mL of PDA medium mixed with 5% v/v Tween 20 (100 μL) were used as control groups. The obtained results were reported as inhibition percentage of mycelial growth: [(mycelial diameter of the untreated—mycelial diameter of treated essential oil)/mycelial diameter of untreated essential oil) × 100]. There were five replicates for each treatment, and the experiment was repeated three times.

### 2.5 Viability test of the treated pathogen

To evaluate the viability of *A*. *aculeatus* after essential oils treatment in *in vitro* test, an agar plug (5 mm) of *A*. *aculeatus* pathogen was transferred to a fresh PDA plate and further cultured at room temperature for seven days. The mycelial growth was determined by measuring their mycelial diameter. This experiment was conducted with five replicates, and repeated three times.

### 2.6 Analysis of the volatile compounds of *C*. *cyminum* essential oil

The volatile compounds of *C*. *cyminum* essential oil was identified using gas chromatograph (HP6890, Agilent Technologies) interfaced to a mass-selective detector (HP model 5973, Agilent Technologies). The columns used was a HP-5MS (5% phenyl polymethylsiloxane) capillary column (30 m × 0.25 mm × 0.25 μm) (Agilent Technologies). Purified helium gas was used as the carrier gas. The temperature program was initially set at 60°C and increased to 220°C at a rate of 3°C/min. Injector temperature was set at 250°C while detector temperature was set at 280°C, respectively. The ionization energy was 70 eV and molecular mass were scanned ranging from 30–300 AMU. The voltage of electron multiplier was programed at 1150 V. The temperature of ion source was set at 230°C while quadrupole temperature was set at 250°C. The volatile compounds were identified by comparing their retention indices relatively to C_8_-C_17_ n-alkanes, with those obtained from Adams databases [27]. Moreover, their spectra were also compared to those found in the Wiley7N, NIST98 Mass Spectral Library. The quantitation of volatile compounds was performed using gas chromatograph (HP 6890, Agilent Technologies) equipped with a flame ionization detector. The volatile components were reported as the relative peak area percentage.

### 2.7 *In vitro* test of pure compounds on the mycelial growth of *A*. *aculeatus*

Antifungal activity of pure volatile compounds including cumin aldehyde and α-terpinen-7-al against *A*. *aculeatus* using the contact phase method described by Aminifard & Mohammadi [25] and Pedrotti et al. [26] with some modifications. These compounds were purchased from Hairui Chemical (China) and various concentrations of 20, 40, 60, 80, and 100 μg/mL were prepared by diluting in 5% v/v Tween 20. After that, 9.0 mL of PDA medium solution was placed into 9 cm diameter Petri dish plates. A 100 μL of each sample was placed at the center of a Petri plate. After five minutes, an agar plug (5 mm) of pathogen *A*. *aculeatus* from seven days precultures was placed at the center of the PDA plate. All plates were sealed with Parafilm and further incubated at room temperature for seven days. Mycelia growth was determined after seven days. The mycelial diameter of *A*. *aculeatus* pathogen treated with pure compounds in the PDA medium and the control group (no pure compound treatment) was measured daily with a digital Vernier caliper (MITUTOYO-ABS Digimatic Caliper CD-AX) in a mm unit. Plates containing 9.0 mL of PDA medium mixed with 5% v/v Tween 20 (100 μL) were used as control groups. The obtained results were reported as inhibition percentage of mycelial growth as explained previously. There were five replicates for each treatment, and the experiment was repeated three times.

### 2.8 Antifungal activity of *C*. *cyminum* essential oil and pure volatile compounds on conidia germination

*C*. *cyminum* essential oil showing strong inhibition percentage in *in vitro* experiments were selected for conidia germination experiments in accordance with the procedure described by Pedrotti et al. [26]. Pure volatile compounds including cumin aldehyde and α-terpinen-7-al were also tested to determine their effect against conidia germination of *A*. *aculeatus*. Conidia of 14-day old *A*. *aculeatus* were used in this experiment. The conidia were slowly poured in 5 mL of sterile water before scraping its surface using a sterile glass rod. The mycelia fragments extracted from the suspensions by filtering through three layers of cheesecloth. Conidial concentration of 1 × 10^6^ conidia/mL was prepared using a hemocytometer under a microscope. A 50 μL conidia suspension was immersed in microtubes containing 500 μL of potato dextrose broth (PDB). Different concentrations of *C*. *cyminum* essential oil (200, 400, 600, 800, and 1,000 μg/mL) or pure volatile compounds (20, 40, 60, 80, and 100 μg/mL) were prepared in 5%v/v Tween 20. PDB, with the addition of 5% v/v Tween 20 was used as the control treatment. All microtubes were further incubated at room temperature for 16 h before transferring samples to a hemocytometer tube. The number of conidia germination was counted under microscope at 10× magnification. EC_50_ values were also calculated as the effective concentration that inhibited conidial germination by 50% in comparison to the control. This experiment was conducted with five replicates, in which 100 conidia were determined in each replicate. This experiment was repeated three times.

### 2.9 *In vivo* antifungal assay of *C*. *cyminum* essential oil and pure volatile compounds

The *in vivo* experiment was performed using the procedure described by Pedrotti et al. [16] with some modifications. All healthy Beauty Seedless grape berries collected from a greenhouse at DoiFang Vineyard Farm (19°51'59.8"N 99°09'25.9"E), Fang district, Chiang Mai province, Thailand located in March 2020. The collection site access has been approved by Ms Sawittree Kaewprasert, the owner of DoiFang Vineyard Farm. They were chosen based on their maturity, size, color without physical infections or injuries. The grape berries were sterilized by washing with distilled water for 2 min followed by 1% sodium hypochlorite for 60 s, and distilled water three times. Those berries were placed on a clean, pre-aseptic bench to air-dry for 30 min before inoculation with the pathogens. One location on each berry was wounded using a sterile inoculating needle. Conidia of pathogen *A*. *aculeatus* was collected from its 14-day old colony in PDA at room temperature with a 12-h photoperiod. The suspension was prepared in sterile water to obtain a suspension concentration of 1 × 10^6^ conidia/mL. The wound depth was approximately 2 mm. In the postharvest curative treatment, twenty healthy grape berries were inoculated simultaneously with 10 μL of a conidia suspension of *A*. *aculeatus*. They were then placed at the bottom of a 2 L plastic box that was covered with sterilized tissue paper. After 4 h, each grape berry was sprayed with *C*. *cyminum* essential oils at various concentrations of 200, 400, 600, 800, and 1,000 μg/mL or pure volatile compounds (cumin aldehyde and α-terpinen-7-al) solutions at various concentrations of 20, 40, 60, 80, and 100 μg/mL in 5%v/v Tween 20. Each box was closed with a fitted plastic lid and air-sealed using Parafilm. All boxes were separated in two groups and then kept at room temperature for 10 and 20 days, respectively. Different clusters of grape berries were prepared for each solution treatment: pathogen-uninoculated (negative control, NC), essential oil-treated (essential oil treatment, TE), cumin aldehyde-treated (cumin aldehyde TE), and α-terpinen-7-al-treated (α-terpinen-7-al TE) grape berries. Groups from NC treatments were treated with *C*. *cyminum* oil at a concentration of 1,000 μg/mL or pure compounds at a concentration of 100 μg/mL, while grape berries in the TE group were treated with *C*. *cyminum* oil and pure volatile compounds at various concentrations. Results from these clusters were compared to those obtained from the clusters without essential oil or pure compound treatments: pathogen-inoculated (positive control, PC) and pathogen-uninoculated (control, CC) grape berries. A digital Vernier caliper (MITUTOYO-ABS Digimatic Caliper CD-AX) was used to measure the mycelial diameter (mm) of *A*. *aculeatus* pathogen detected in each sample. Each treatment was conducted with five replicates. This experiment was repeated three times. *In vivo* antifungal effect was expressed in terms of disease incidence and severity percentage. Disease incidence was calculated as [(numbers of diseased fruit/total Number of fruit)×100]. Disease severity was calculated using the following formula: [(sum of symptomatic fruits and their corresponding score scale)/(total number of fruits × highest score scale)]×100. The severity scale was considered from 0 to 100% according to Pedrotti et al. [26].

### 2.10 Quality of grape berries obtained from all treatments

The quality of Beauty Seedless grape berries was evaluated after treatments with *C*. *cyminum* essential oil, cumin aldehyde, and α-terpinen-7-al for 10 and 20 days. In this experiment, weight loss percentage, fruit firmness, total soluble solids, and pH were measured following the study by Jahani et al. [28]. Total phenolic content and antioxidant activity of grape berries after treatment were determined following the study by Molyneux [29] and Yao et al. [30]. Each experiment was conducted with five replicates, and repeated three times.

#### Weight loss percentage

Weight loss of grape berries was determined as the percentage of weight loss with the following formula: (Initial weight—terminal weight)/(terminal weight) × 100.

#### Fruit firmness

Fruit firmness of samples was measured using a TA-XT Plus texture analyzer (Stable Micro Systems Ltd., UK).

#### Total soluble solids

Total soluble solids of the grape juices were determined at room temperature using a digital refractometer (RF 10, 0–32° Brix, Extech Co., USA).

#### pH

The pH value of grape juices was measured at room temperature using a pH meter (Thermo Scientific Orion Star A111 Benchtop pH Meter, USA).

#### Total phenolic content and antioxidant activity

Grape berries from all treatments were homogenized and centrifuged at 13,000 rpm at room temperature for 10 min to extract their bioactive compounds. The obtained supernatants were used to be determined their phenolic content and antioxidant activity.

For the total phenolic content assay, extract (100 μL) was mixed with phosphate buffer (400 μL) and 10% v/v Folin-Ciocalteu phenol reagent (5 mL). After 1 min, 4 mL of 7.5% w/v sodium carbonate solution was added to the mixture and further kept in a dark place at room temperature for 1 h. Absorbance spectrum of the solution was determined using a UV/Vis spectrophotometer (Perkin-Elmer-Lamda 25) at 765 nm. Gallic acid was used as a standard.

Antioxidant activity of grape berries extracts was analyzed using 2,2-diphenyl-1-picrylhydrazyl (DPPH) assay. Grape extract (1 mL) was added to 60 mM DPPH methanolic solution (1 mL). The solution was vigorously mixed and stored in a dark place at room temperature for 30 min. Absorbance of all solutions were determined using UV/Vis spectrophotometer (Perkin-Elmer-Lamda 25) at 517 nm. Trolox was used as a standard.

### 2.11 Data analysis

The differences in all experiments were analyzed using analysis of variance (ANOVA), using the SPSS 20.0 software. Duncan’s post-hoc test was used to compare the mean values. A significance level at P < 0.05 was set to determine the significant differences. All data were reported as mean values with standard deviations.

## 3. Results

### 3.1 *In vitro* antifungal activity of essential oils on the *A*. *aculeatus* mycelial growth

*Z*. *bungeanum* Maxim., *C*. *sativum*, *Z*. *rhetsa*, *C*. *cyminum*, *C*. *sativum*, and *Z*. *montanum* (J. Koenig) Link ex A. Dietr. presented essential oil yields of 0.28%, 0.48%, 0.57%, 0.89%, and 0.93%, respectively. All essential oils were screened *in vitro* antifungal activity against *A*. *aculeatus* on mycelial growth using contact phase assay. The effects of different essential oil concentrations on the *A*. *aculeatus* mycelial growth are listed in Table 1. The inhibition rate of essential oils on the *A*. *aculeatus* mycelial growth was most prominent at day 1, while the lowest was observed at day 7. A significant inhibition was observed until the day 7 compared to the control. High inhibition rate (>80%) was observed in *C*. *cyminum* essential oil from a concentration of 800 and 1,000 μg/mL at all days while other essential oils showed low inhibition rate (<50%) at most concentrations and number of days. Therefore, *C*. *cyminum* essential oils had significantly highest inhibition rate and were chosen for further tests.

**Table 1:** Antifungal activity of *C*. *cyminum*, *Z*. *bungeanum* Maxim., *C*. *sativum*, *Z*. *montanum* (J. Koenig) Link ex A. Dietr., and *Z*. *rhetsa* essential oils at different concentrations against *A*. *aculeatus* using contact phase assay.

Essential oil	Concentration (μg/mL)	% inhibition						
		Day1	Day2	Day3	Day4	Day5	Day6	Day7
*C*. *cyminum*	200	78.27±0.28 e	76.44±1.09 e	76.37±0.33 e	72.66±0.58 e	72.64±0.98 e	70.41±0.83 e	69.69±1.68 d
	400	82.06±0.81 d	80.79±0.30 d	80.05±0.50 d	79.27±0.60 d	79.08±0.27 d	76.19±1.13 d	75.76±0.76 c
	600	85.04±0.23 c	84.13±0.53 c	83.75±0.93 c	83.43±0.28 c	82.43±0.47 c	79.87±0.90 c	76.13±1.49 b
	800	91.09±0.34 b	90.23±0.25 b	87.05±0.56 b	86.09±1.12 b	85.49±0.43 b	82.55±0.90 b	81.48±0.66 b
	1,000	95.08±0.12 a	92.25±0.38 a	89.29±0.51 a	87.64±0.52 a	87.20±0.85 a	86.04±0.57 a	82.13±1.49 a
*Z*. *bungeanum*	200	38.52±2.00 d	34.09±0.83 e	31.47±1.26 e	27.49±0.00 e	25.57±1.15 e	24.21±0.00 e	22.49±1.01 e
	400	39.26±0.98 d	36.94±1.29 d	33.87±1.42 d	31.03±1.13 d	29.57±1.00 d	28.91±0.96 d	26.91±0.94 d
	600	44.97±1.81 c	40.65±1.42 c	39.29±1.35 c	37.83±1.77 c	36.31±0.88 c	35.05±1.46 c	33.46±0.84 c
	800	47.72±1.79 b	44.69±1.31 b	43.03±1.05 b	41.75±1.61 b	39.10±1.69 b	37.25±0.82 b	35.05±1.42 b
	1,000	50.39±198 a	47.73±1.42 a	45.20±1.66 a	43.66±0.95 a	41.82±1.37 a	41.22±0.52 a	39.10±0.53 a
*C*. *sativum*	200	41.27±0.68 e	29.77±0.40 e	27.18±1.08 e	26.94±0.54 e	23.62±0.55 d	21.69±0.98 d	21.39±1.20 c
	400	45.85±0.54 d	34.22±0.39 d	30.62±0.91 d	30.54±1.45 d	22.79±0.92 c	21.94±0.93 c	21.11±1.64 b
	600	47.84±0.64 c	46.64±1.07 c	43.60±0.51 c	43.25±1.42 c	31.52±0.90 b	29.40±0.53 b	22.50±0.67 a
	800	50.98±0.88 b	49.06±0.74 b	47.44±0.37 b	45.13±0.38 b	31.19±1.34 a	31.22±0.80 a	28.52±1.27 a
	1,000	53.38±0.30 a	51.00±0.47 a	50.06±0.37 a	48.74±0.58 a	47.65±0.98 a	36.37±0.87 a	36.00±0.75 a
*Z*. *montanum*	200	40.13±0.56 d	39.29±1.32 e	37.25±0.54 d	26.47±1.64 d	24.74±1.09 d	23.65±0.66 d	23.14±0.96 c
	400	43.66±0.95 d	42.00±2.06 d	41.91±1.70 c	28.79±0.92 d	25.05±0.95 c	24.03±1.32 c	23.78±0.55 b
	600	46.46±0.92 c	44.24±1.33 c	43.31±0.89 b	43.00±1.82 c	30.31±1.39 b	28.18±0.53 b	25.16±1.74 a
	800	50.97±1.54 b	49.06±0.97 b	45.56±1.31 b	44.48±2.18 b	42.71±1.03 a	40.62±0.91 a	40.55±1.13 a
	1,000	51.86±0.88 a	51.51±0.59 a	50.72±0.48 a	48.78±0.49 a	47.52±1.12 a	45.64±0.50 a	45.12±1.29 a
*Z*. *rhetsa*	200	46.16±0.54 d	45.56±2.16 d	33.36±0.77 e	32.56±0.54 c	14.74±0.55 e	12.50±0.56 e	12.11±1.64 e
	400	48.75±0.64 c	46.63±2.13 c	44.24±1.33 d	31.29±1.13 b	27.40±0.93 d	24.78±1.73 d	24.58±0.55 d
	600	49.19±1.27 b	48.75±1.82 b	45.79±0.38 c	45.50±0.61 b	40.16±0.64 c	26.94±0.66 c	26.29±1.72 c
	800	52.29±1.85 b	51.51±0.29 a	48.07±0.49 b	46.86±2.23 a	44.63±0.51 b	31.97±0.52 b	28.52±1.27 b
	1,000	54.16±0.98 a	53.52±0.58 a	51.10±0.72 a	50.45±0.48 a	47.36±1.31 a	44.62±1.60 a	42.42±0.89 a

Data are mean ± standard deviation. Different letters indicate significant difference within each essential oil concentrations determined in each day according to Duncan’s post-hoc test (P<0.05).

### 3.2 Viability of the treated pathogen

**Fig 1:**
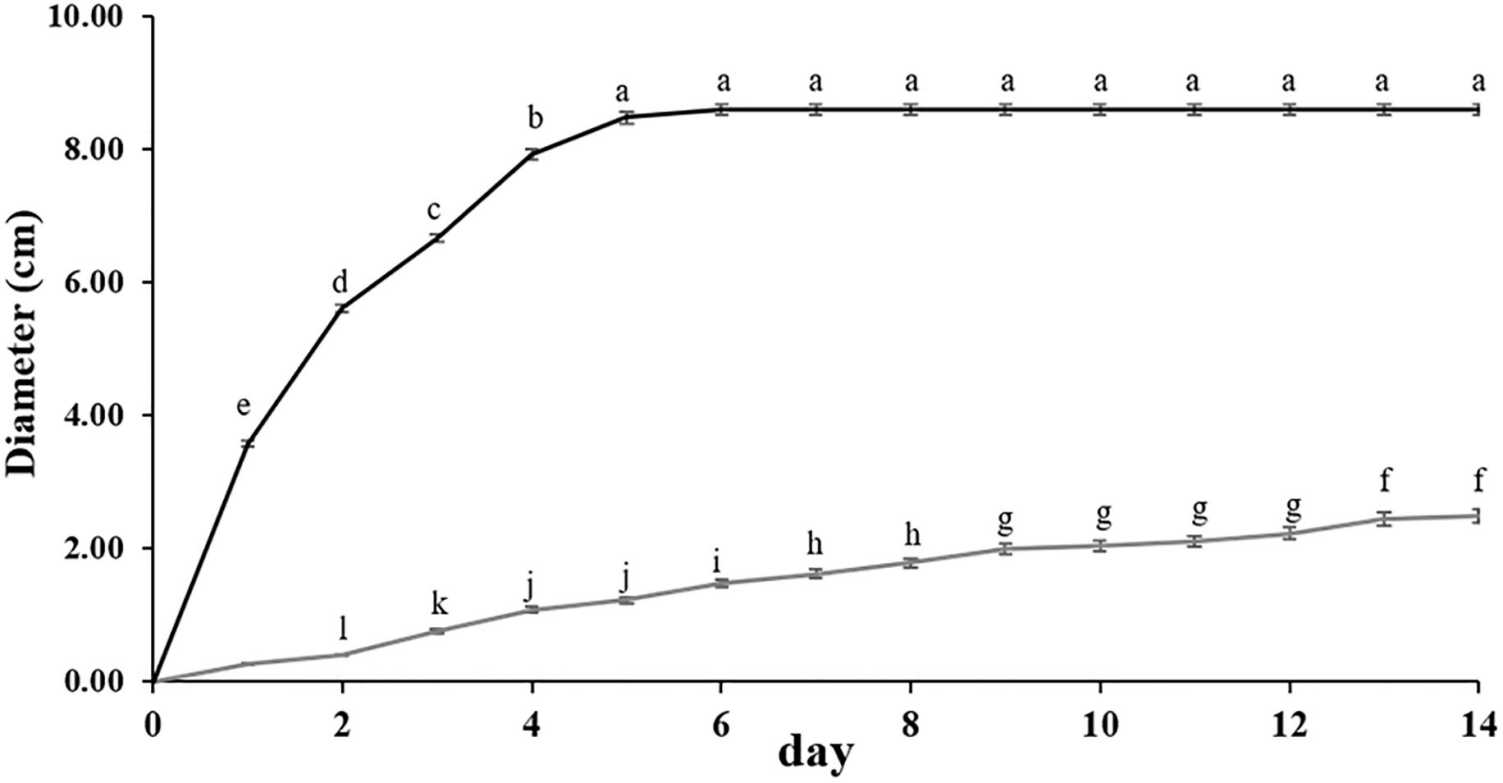
Diameter of *A*. *aculeatus* pathogen after treating (■) and without treating (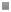) with *C*. *cyminum* essential oil on medium agar plates. Data are mean ± standard deviation. Different letters indicate significant difference within each essential oil concentrations determined in each day according to Duncan’s post-hoc test (P<0.05).

After the treatment of *A*. *aculeatus* with the essential oils *C*. *cyminum*, pathogen *A*. *aculeatus* was able to grow and produce its mycelia slowly again on the PDA plates. The diameters of *A*. *aculeatus* pathogen after treating and without treating with *C*. *cyminum* essential oil on medium agar plates are shown in Fig 1. The growth of *A*. *aculeatus* after treating with *C*. *cyminum* essential oil was significantly slower than those obtained from untreated pates. After 14 days, diameter of *A*. *aculeatus* pathogen after treating and without treating with *C*. *cyminum* was 2.51 cm and 8.63 cm, respectively.

### 3.3 Analysis of volatile compounds of *C*. *cyminum* essential oil

GC-MS chromatogram of *C*. *cyminum* oil is presented in S1 Fig. Volatile compounds of *C*. *cyminum* oil are listed in Table 2. Twenty-four components were identified in *C*. *cyminum* essential oil, comprising 100% of the essential oil. The major compounds in *C*. *cyminum* oil were cumin aldehyde (33.94%), α-terpinen-7-al (32.20%), γ-terpinen-7-al (13.74%), γ-terpinene (6.67%), β-pinene (5.34%) and p-cymene (3.58%).

**Table 2:** Volatile compounds of *C*. *cyminum* essential oil.

No.	Compound	RI	% Relative area
**Monoterpene Hydrocarbons**			
1	Tricyclene	921	0.11
2	α-Thujene	924	0.25
3	α-Pinene	932	0.37
4	β-Pinene	974	5.34
5	Myrcene	988	0.39
6	α-Phellandrene	1002	0.06
7	γ-carene	1008	0.02
8	α-Terpinene	1014	0.06
9	p-Cymene	1020	3.58
10	Limonene	1024	0.22
11	γ-Terpinene	1054	6.67
12	Terpinolene	1086	0.03
**Oxygenated monoterpenes**			
13	1,8-Cineole	1026	0.15
14	cis-Sabinene hydrate	1065	0.06
15	trans-Sabinene hydrate	1098	0.10
16	cis-p-Menth-2-en-1-ol	1118	0.04
17	trans-p-menth-2-en-1-ol	1136	0.10
18	cis-β-Terpineol	1140	0.22
19	α-Terpineol	1186	0.13
20	Pulegone	1233	1.58
21	Cumin aldehyde	1238	33.94
22	γ-Terpinen-7-al	1260	13.74
23	α-Terpinen-7-al	1290	32.20
24	p-Mentha-1,4-dien-7-ol	1325	0.65

### 3.4 *In vitro* test of pure compound on the mycelial growth of *A*. *aculeatus*

All concentrations of pure major volatile compounds of *C*. *cyminum* oil (cumin aldehyde and α-terpinen-7-al) were tested to determine their antifungal activity against *A*. *aculeatus* using contact phase assay that showed in Table 3. The reduction on mycelial growth of *A*. *aculeatus* was most prominent at day 1 and continuously reduced at day 7. High inhibition rate (>80%) was recorded significantly in cumin aldehyde at concentration 60, 80, and 100 μg/mL, respectively, at day 1. Meanwhile, at all concentrations and number of days of α-terpinen-7-al showed low inhibition rate (<70%) at most concentrations and number of days. Hence, cumin aldehyde had significant inhibition rate on the mycelial growth of *A*. *aculeatus*.

**Table 3:** Antifungal activity of cumin aldehyde and α-terpinen-7-al at different concentrations against *A*. *aculeatus* using contact phase assay.

Essential oil	Concentration (μg/mL)	% inhibition						
		Day1	Day2	Day3	Day4	Day5	Day6	Day7
Cumin aldehyde	20	72.04±0.85 e	69.55±1.52 d	62.61±0.98 d	60.90±1.00 d	58.57±1.02 d	56.77±1.04 d	54.94±1.07 c
	40	77.13±0.76 d	71.06±0.00 d	70.55±1.71 c	64.81±1.64 c	61.46±1.71 c	59.16±1.02 c	56.16±1.83 c
	60	80.11±0.71 c	74.42±0.80 c	72.04±0.85 c	69.55±1.52 b	66.44±0.00 b	63.16±1.67 b	60.90±1.00 b
	80	82.10±0.67 b	78.86±0.74 b	74.88±0.80 b	71.55±0.85 b	68.53±0.89 b	64.27±0.96 b	62.04±0.98 b
	100	85.07±1.07 a	81.32±0.69 a	78.43±0.74 a	75.33±1.37 a	72.52±1.45 a	68.53±0.89 a	65.36±0.93 a
α-terpinen-7-al	20	54.94±1.07 e	51.16±1.11 e	48.55±1.98 d	45.88±1.18 e	41.04±1.22 e	38.18±2.17 d	33.01±2.59 d
	40	59.16±1.02 d	55.55±1.07 d	51.80±1.11 c	49.21±1.13 d	47.23±1.15 d	43.14±1.20 c	39.62±1.24 c
	60	62.61±0.98 c	60.32±1.00 c	57.37±1.04 b	53.70±1.09 c	50.52±0.00 c	47.23±1.15 b	43.82±2.37 b
	80	66.97±0.91 b	63.72±0.96 b	59.16±1.02 b	56.16±1.83 b	53.07±1.09 b	49.21±1.13 b	45.20±1.18 ab
	100	71.55±0.85 a	67.49±0.91a	63.72±0.96 a	59.16±1.02 a	55.55±1.07 a	51.80±1.11 a	47.89±1.15 a

Data are mean ± standard deviation. Different letters indicate significant difference within each essential oil concentrations determined in each day according to Duncan’s post-hoc test (P<0.05).

### 3.5 Antifungal activity of *C*. *cyminum* essential oil and pure volatile compounds on conidia germination

**Fig 2:**
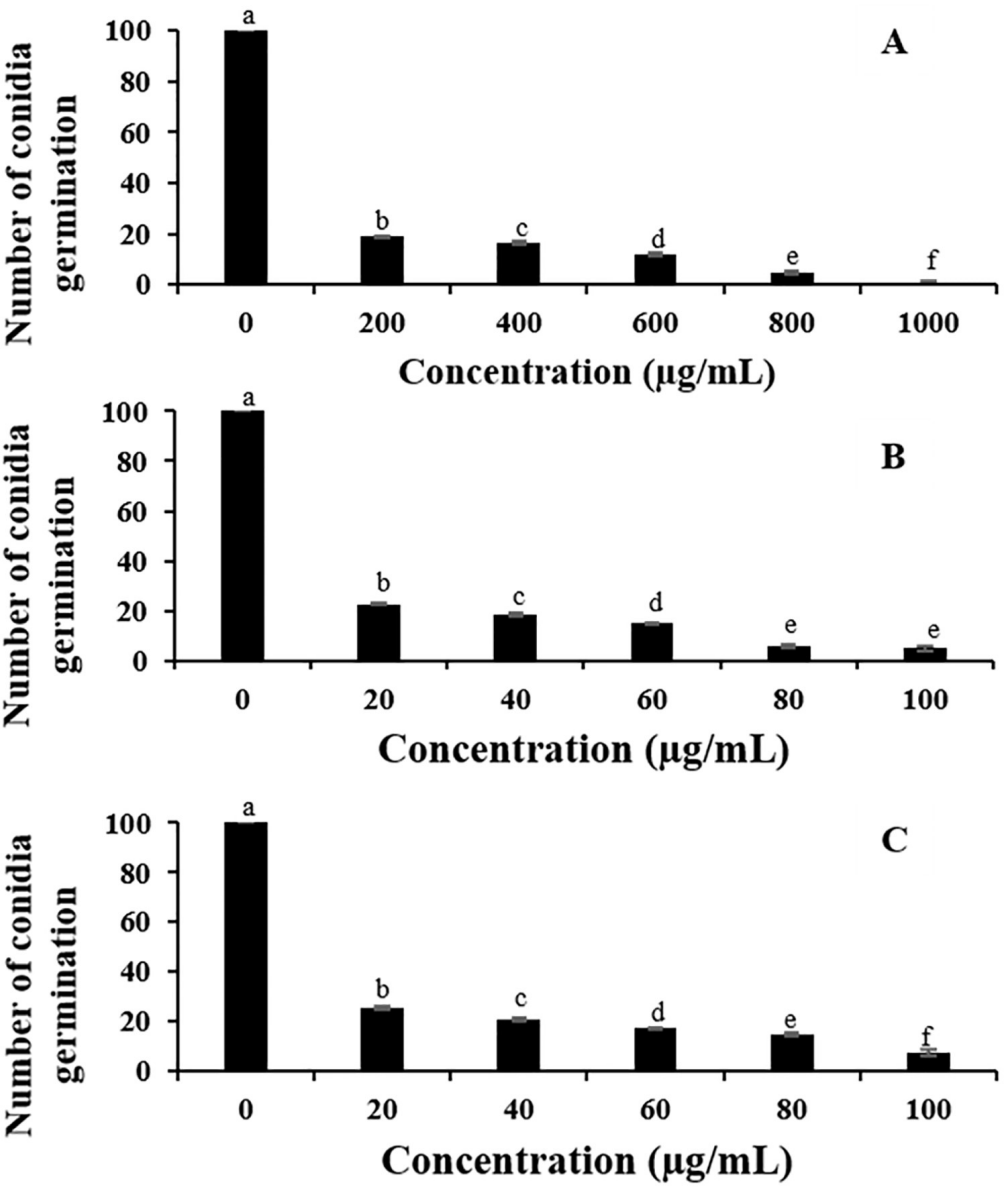
Effect of different concentrations of *C*. *cyminum* essential oil (A), cumin aldehyde (B), and α-terpinen-7-al (C) on conidia germination of *A*. *aculeatus*. Data are mean ± standard deviation. Different letters indicate significant difference within each essential oil concentrations determined in each day according to Duncan’s post-hoc test (P<0.05).

The inhibitory effect of *C*. *cyminum* essential oil and pure volatile compounds from different concentrations on conidia germination of *A*. *aculeatus* is depicted in Fig 2. The conidia germination of *A*. *aculeatus* was significantly lower in the highest concentration. At all concentrations of *C*. *cyminum* essential oil, cumin aldehyde and α-terpinen-7-al, a significant reduction of conidia germination was observed in comparison with the control group. *C*. *cyminum* essential oil and cumin aldehyde inhibited conidia germination of *A*. *aculeatus* at the highest concentration (1000 and 100 μg/mL), remaining 1.0 and 4.8 conidia germinated, respectively, while α-terpinen-7-al showed significant reduction among concentration 60–100 μg/mL. EC_50_ values of the *C*. *cyminum* essential oil, cumin aldehyde, and α-terpinen-7-al were 67.28 μg/mL, 9.31 μg/mL, and 13.23 μg/mL, respectively.

### 3.6 *In vivo* antifungal assay of *C*. *cyminum* essential oil, cumin aldehyde, and α-terpinen-7-al

**Fig 3:**
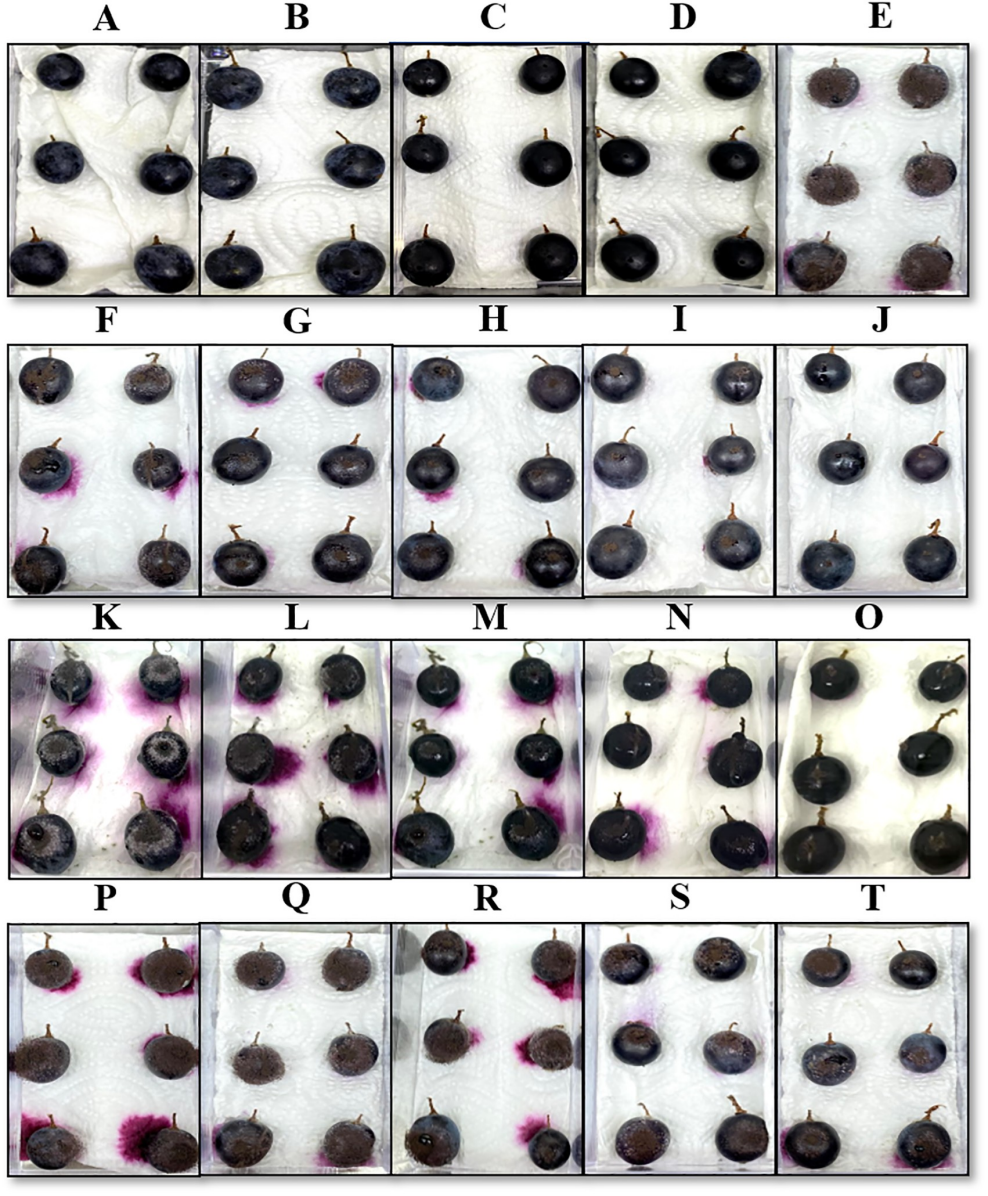
*In vivo* assay of the antifungal activity of the *C*. *cyminum* essential, cumin aldehyde, and α-terpinen-7-al against *A*. *aculeatus* infections after 10 days. Grape berries obtained from various treatments: CC (A), *C*. *cyminum* NC (B), cumin aldehyde NC (C), α-terpinen-7-al NC (D), PC (E), *C*. *cyminum* TE (F-J) at concentrations of 200, 400, 600, 800 and 1,000 μg/mL, cumin aldehyde TE (K-O) and α-terpinen-7-al TE (P-T) at concentrations of 20, 40, 60, 80 and 100 μg/mL, respectively.

**Fig 4:**
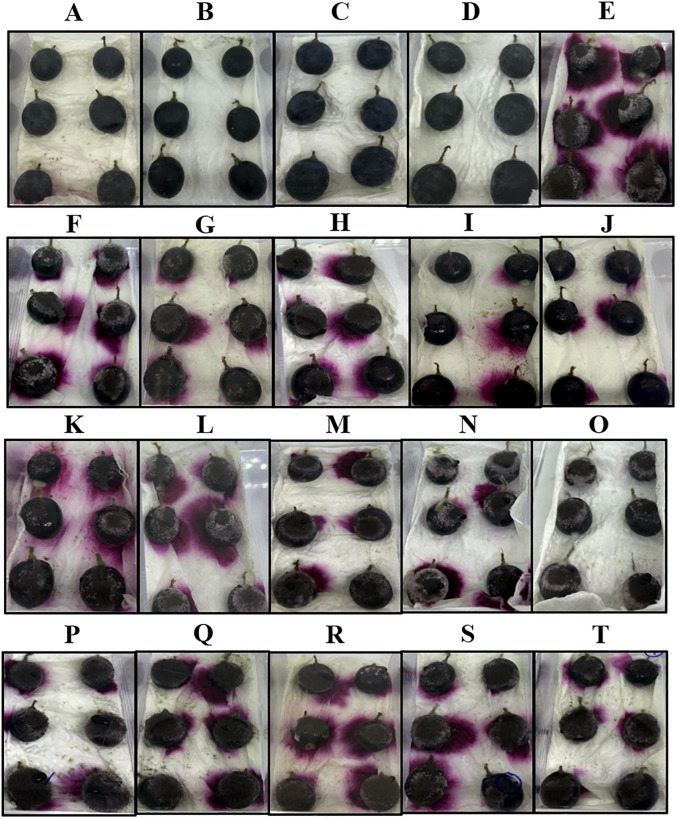
*In vivo* assay of the antifungal activity of the *C*. *cyminum* essential, cumin aldehyde, and α-terpinen-7-al against *A*. *aculeatus* infections after 20 days. Grape berries obtained from various treatments: CC (A), *C*. *cyminum* NC (B), cumin aldehyde NC (C), α-terpinen-7-al NC (D), PC (E), *C*. *cyminum* TE (F-J) at concentrations of 200, 400, 600, 800 and 1,000 μg/mL, cumin aldehyde TE (K-O) and α-terpinen-7-al TE (P-T) at concentrations of 20, 40, 60, 80 and 100 μg/mL, respectively.

**Fig 5:**
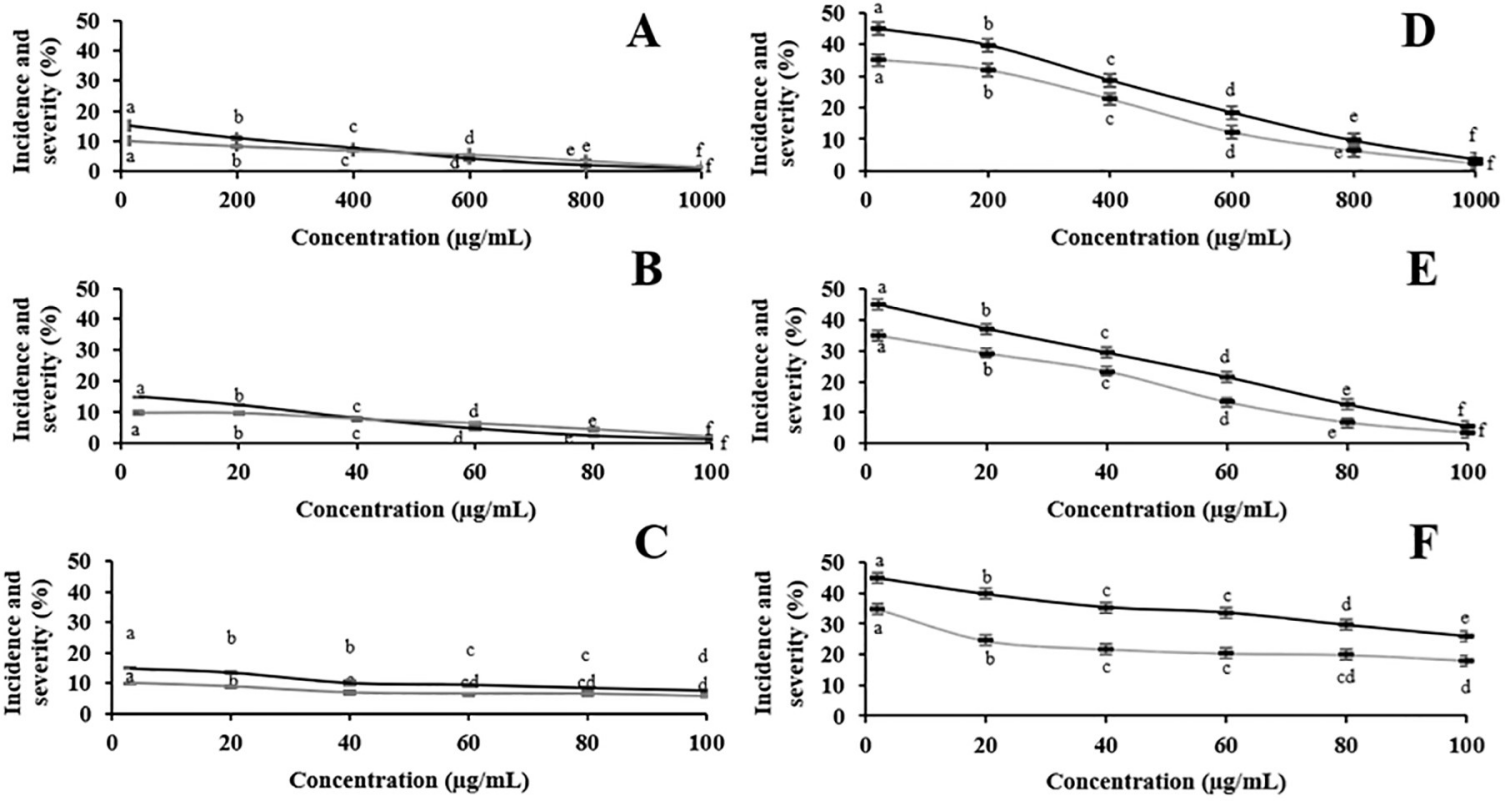
Severity (■) and incidence (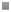) percentage of *A*. *aculeatus* in grape berries treated with different concentrations of *C*. *cyminum* essential oil (A and D), cumin aldehyde (B and E), and α-terpinen-7-al (C, F) after 10 days and 20 days, respectively. Data are mean ± standard deviation. Different letters indicate significant difference within each essential oil concentrations determined in each day according to Duncan’s post-hoc test (P<0.05).

Grape berries after 10- and 20-day incubation from various treatments are shown in Figs 3 and 4, respectively. No physical damage on the grape berries was detected in the CC and NC treatments among all treatments. Less physical damage was observed on grape berries inoculated with *A*. *aculeatus* and treated with *C*. *cyminum* oil (≥800 μg/mL) and cumin aldehyde (100 μg/mL) after 10-day incubation. Moderate physical damage was observed on grape berries inoculated with *A*. *aculeatus* and treated with *C*. *cyminum* oils at concentrations of ≤600 μg/mL, cumin aldehyde at concentrations of ≤80 μg/mL, and α-terpinen-7-al at all concentrations after 10-day incubation. However, high physical damage was observed on grape berries inoculated with *A*. *aculeatus* and treated with all concentrations after 20-day incubation. Incidence and severity percentage of *A*. *aculeatus* in grape berries treated with *C*. *cyminum* oil, cumin aldehyde, and α-terpinen-7-al from various concentrations after 10- and 20-day incubation are shown in Fig 5. Significant reduction of the incidence and severity percentage was observed in all treatments. After 10-day incubation, *C*. *cyminum* oil (1,000 μg/mL) and cumin aldehyde (100 μg/mL) treatments showed the highest reduction among these concentrations with 1.48% incidence and 0.69% severity for *C*. *cyminum* oil and 2.04% incidence and 1.11% severity for cumin aldehyde while no significant reduction of the incidence and severity percentage was observed in α-terpinen-7-al treatment at concentration of 60 μg/mL. After 20-day incubation, the highest reduction of incidence and severity percentage was also detected from the treatment with *C*. *cyminum* oil followed by cumin aldehyde, and α-terpinen-7-al at the highest concentration with % incidence of 3.70, 5.56, and 25.93, and % severity of 2.59, 3.33, and 18.06, respectively.

### 3.7 Quality of grape berries after treatments

The effects of *C*. *cyminum* oil, cumin aldehyde, and α-terpinen-7-al essential oils on the quality of grape berries after 10- and 20-day incubation in terms of weight loss percentage, firmness, total soluble solids, pH, total phenolic content, and antioxidant activity are depicted in Tables 4 and 5, respectively. After ten days of treatments, no significant difference was observed for pH of the grape berries from all treatments (3.24–3.43) while other qualities including % weight loss, firmness, total phenolic content, antioxidant activity and total soluble solids were significance among these treatments. Weight loss percentage, firmness, total phenolic content, antioxidant activity and total soluble solids among these treatments were ranged from 1.53–3.34%, 0.31–0.51 N, 3.92–10.28 mg GAE/g oil, 18.63–43.51 μM Trolox/g oil, 17.70–20.22°Brix, respectively. Furthermore, treatments by *C*. *cyminum* essential oil and cumin aldehyde did not have a negative effect on grape quality. After 20 days of treatments, similar pH values were detected with on significance from all treatments (3.22–3.42) while other qualities were significance among these treatments. Weight loss percentage, firmness, total phenolic content, antioxidant activity and total soluble solids among these treatments were ranged from 3.88–8.39%, 0.16–0.24 N, 1.89–4.79 mg GAE/g oil, 8.50–20.46 μM Trolox/g oil, 16.50–19.01°Brix, respectively.

**Table 4:** Quality of grape berries obtained from all treatments after 10 days.

Treatments		% Weight loss	Firmness (N)	Total phenolic content (mg GAE/g oil)	Antioxidant activity (μM Trolox/g oil)	pH	TSS (°Brix)
CC		2.08±0.01 hi	0.45±0.01 cd	7.07±0.01 k	29.83±0.07 j	3.41±0.00 ab	19.66±0.03 bc	
PC		3.32±0.02 o	0.31±0.01 i	3.92±0.01 l	18.63±0.07 l	3.24±0.01 f	17.70±0.19 g
*C*. *cyminum* NC		2.11±0.01 j	0.41±0.01 ef	7.33±0.03 j	27.42±0.23 k	3.42±0.01 ab	18.63±0.06 e
Cumin aldehyde NC		2.08±0.01 i	0.42±0.01 de	7.38±0.01 j	28.40±0.51 k	3.41±0.02 ab	18.83±0.10 e
α-terpinen-7-al NC		2.06±0.01 h	0.40±0.01 f	7.35±0.03 j	28.55±0.09 k	3.42±0.02 ab	18.87±0.05 e
*C*. *cyminum* TE							
concentration (μg/mL)	200	2.25±0.01 l	0.46±0.03 bc	9.52±0.03 d	38.87±0.11 c	3.42±0.01 ab	19.28±0.07 d
	400	1.96±0.01 g	0.47±0.02 b	9.54±0.02 d	38.32±0.03 cd	3.42±0.03 ab	19.24±0.14 d
	600	1.84±0.01 e	0.47±0.02 bc	9.67±0.02 cd	39.56±0.31 bc	3.42±0.01 ab	18.77±0.08 e
	800	1.95±0.01 g	0.48±0.01 b	9.87±0.03 b	40.62±0.33 b	3.43±0.03 a	18.72±0.23 e
	1,000	1.77±0.01 c	0.51±0.01 a	10.28±0.03 a	43.51±0.26 a	3.43±0.03 a	18.71±0.04 e
Cumin aldehyde TE concentration (μg/mL)	20	2.06±0.02 h	0.44±0.01 d	8.45±0.16 g	35.28±0.80 fg	3.42±0.01 ab	18.29±0.17 f
	40	1.81±0.02 d	0.44±0.01 cd	8.69 ±0.01 f	36.45±1.79 ef	3.42±0.03 ab	18.64±0.50 e
	60	1.86±0.01 f	0.45±0.01 cd	8.38±0.38 gh	36.95±1.85 e	3.42±0.01 ab	18.70±0.11 e
	80	1.66±0.01 b	0.46±0.01 bc	9.79±0.01 bc	37.11±0.63 de	3.43±0.03 a	19.40±0.34 cd	
	100	1.53±0.01 a	0.48±0.01 b	9.84±0.01 b	37.35±1.48 de	3.43±0.03 a	19.27±0.39 d
α-Terpinen-7-al TE							
concentration (μg/mL)	20	3.34±0.01 o	0.34±0.03 h	7.85±0.06 i	32.93±0.21 i	3.33±0.02 e	19.71±0.20 bc
	40	3.22±0.01 n	0.35±0.01 h	7.76±0.02 i	33.54±0.19 hi	3.37±0.01 cd	19.77±0.03 b
	60	2.15±0.01 k	0.36±0.01 gh	8.26±0.03 h	33.88±0.08 hi	3.35±0.01 de	20.12±0.04 a
	80	2.30±0.01 m	0.35±0.02 h	9.26±0.03 e	34.27±0.25 gh	3.37±0.02 cd	20.20±0.10 a
	100	2.16±0.01 k	0.38±0.01 g	9.55±0.05 d	34.65±0.12 gh	3.39±0.01 bc	20.22±0.08 a

Data are mean ± standard deviation. Different letters indicate significant difference within each group according to Duncan’s post-hoc test (P<0.05).

**Table 5:** Quality of grape berries obtained from all treatments after 20 days.

Treatments		% Weight loss	Firmness (N)	Total phenolic content (mg GAE/g oil)	Antioxidant activity (μM Trolox/g oil)	pH	TSS (°Brix)
CC		5.08±0.13 h	0.21±0.00 bc	3.30±0.01 g	13.88±0.00 i	3.39±0.01 cd	18.39±0.28 bcd
PC		8.39±0.22 r	0.13±0.01 g	1.89±0.00 h	8.50±0.02 j	3.22±0.01 h	16.50±0.22 h
*C*. *cyminum* NC		5.16±0.48 i	0.19±0.01 bcd	3.46±0.01 de	12.91±0.02 i	3.40±0.01 bc	17.58±0.14 efg
Cumin aldehyde NC		5.28±0.09 j	0.19±0.02 bcd	3.44±0.01 fg	13.38±0.01 i	3.40±0.01 bc	17.75±0.16 def
α-terpinen-7-al NC		5.36±0.08 k	0.17±0.04 cde	3.42±0.01 fg	13.51±0.02 i	3.39±0.01 cd	17.69±0.14 def
*C*. *cyminum* TE							
concentration (μg/mL)	200	5.69±0.26 n	0.21±0.01 bc	4.41±0.02 b	18.19±0.01 bcd	3.39±0.02 a	17.57±0.11 efg
	400	5.09±0.19 h	0.21±0.01 ab	4.43±0.02 b	17.90±0.01 bcd	3.40±0.01 a	17.60±0.22 efg
	600	4.76±0.49 f	0.22±0.01 ab	4.49±0.03 b	18.50±0.01 bc	3.40±0.01 ab	17.64±0.21 efg
	800	4.91±0.20 g	0.22±0.01 ab	4.60±0.02 ab	18.99±0.01 b	3.42±0.02 a	17.98±0.24 cde
	1,000	4.37±0.16 c	0.24±0.01 a	4.79±0.01 a	20.46±0.02 a	3.42±0.02 a	18.06±0.26 cde
Cumin aldehyde TE concentration (μg/mL)	20	5.09±0.11 h	0.20±0.01 bcd	3.94±0.01 cd	16.50±0.01 fgh	3.38±0.01 d	17.14±0.33gh
	40	4.51±0.25 d	0.20±0.02 bcd	4.06±0.02 c	17.08±0.02 efg	3.39±0.01 cd	17.43±0.34 fg
	60	4.68±0.28 e	0.21±0.01 bc	4.02±0.01 cd	17.24±0.02 def	3.39±0.01cd	17.47±0.33 efg
	80	4.24±0.39 b	0.21±0.01 bc	4.57±0.01 ab	17.35±0.01 def	3.40±0.01 bc	18.17±0.25 cde
	100	3.88±0.15 a	0.22±0.011 ab	4.59±0.02 ab	17.70±0.01 cde	3.40±0.00 bc	18.22±0.30 bcd
α-Terpinen-7-al TE							
concentration (μg/mL)	20	8.21±0.15 q	0.16±0.02 f	3.65±0.02 ef	15.44±0.01 h	3.30±0.01 g	18.40±0.18 bcd
	40	8.01±0.44 p	0.17±0.01 ef	3.62±0.02 ef	15.65±0.01 h	3.35±0.01 f	18.47±0.21 abc
	60	5.84±0.29 o	0.17±0.02 f	3.84±0.04 cde	15.82±0.02 gh	3.34±0.01 f	18.83±0.18 ab
	80	5.65±0.36 m	0.18±0.02 cde	4.35±0.02 b	16.06±0.02 fgh	3.35±0.01 ef	18.90±0.22 ab
	100	5.42±0.47 l	0.18±0.03 cde	4.47±0.02 b	16.17±0.02 fgh	3.36±0.01 e	19.01±0.31 a

Data are mean ± standard deviation. Different letters indicate significant difference within each group according to Duncan’s post-hoc test (P<0.05).

## 4. Discussion

Essential oils from five plant species were selected for this study due to their broad-spectrum antimicrobial activity. According to previous studies, these essential oils were reported to produce volatile compounds showing antimicrobial activity against *Aspergillus niger* and *Candida* species [31, 32]. Moreover, they have been evaluated to inhibit some phytopathogens such as *Penicillium lilacinum*, *A*. *niger*, *A*. *flavus* and *Botrytis cinerea* [33–36]. Therefore, essential oils extracted from these plant species may be capable of producing volatile compounds that suppress the bunch rot disease caused by *A*. *aculeatus* in postharvest grapes.

Among these essential oils, *C*. *cyminum* essential oil showed high antifungal activity inhibiting the *A*. *aculeatus* mycelial growth and its infections on postharvest grapes. Based on *in vitro* assay, the volatile compounds produced by *C*. *cyminum* essential oil only showed high antifungal activity and their effectiveness gradually decreased over time (Table 1). This result was good agreement to the reports [36, 37] in which essential oils of *C*. *cyminum* oil showed potent inhibitory activity against *A*. *fumigatus*. In this study, *C*. *cyminum* essential oil was effective in controlling *A*. *aculeatus* at all concentrations, while low concentration of *C*. *sativum*, *Z*. *montanum* (J. Koenig) Link ex A. Dietr., *Z*. *bungeanum* Maxim., and *Z*. *rhetsa* essential oils (200 and 400 μg/mL) were sufficient to control *A*. *aculeatus*.

Stronger inhibitory effect on mycelial growth and conidia germination of *A*. *aculeatus* were observed in *C*. *cyminum* oil than the pure volatile compounds treatment. This result was similar to the study from Edris & Farrag [38] reporting high antifungal activity of sweet basil essential oil compared to those obtained by pure major compounds. Moreover, Cavaleiro et al. [39] also reported that *Juniperus* essential oil showed greater antimicrobial activity that those found in their major compounds such as α-pinene, β-phellandrene, and myrcene.

The *in vivo* study, after 10 days of storage at room temperature, showed that treatments by *C*. *cyminum* essential oil at 1,000 μg/mL effectively reduced incidence and severity percentage of *A*. *aculeatus* infection in grape berries compared to other treatments from 10 and 20 days incubation. Previous studies have reported that *C*. *cyminum* oil could control or inhibit phytopathogens such as *B*. *cinerea*, *Penicillium italicum*, *P*. *expansum*, *P*. *commune*, *Rhizopus stolonifer*, and *R*. *lyococcus* in postharvest products [40, 41]. In addition, the mycelial growth of *A*. *aculeatus* was inhibited by *Cinnamomum zeylanicum*, *Ocimum gratissimum*, *Syzygium aromaticum* and *Eugenia caryophyllata* essential oils [42–44]. *In vivo* effectivity of *C*. *cyminum* essential oil was probably due to the antifungal activity of their volatile compounds. Generally, complexity of the essential oil composition has been reported to influence its antimicrobial property [45]. Antifungal activity of the *C*. *cyminum* essential oil may be attributed to cumin aldehyde and α-terpinen-7-al [46, 47]. According to previous studies, cumin aldehyde has been reported to inhibit *Colletotrichum lindemuthianum*, *Fusarium oxysporum*, and *Varticillium dahliae* pathogens while α-terpinen-7-al showed antifungal activities against *Candida* spices [47–49]. The antifungal effect of *C*. *cyminum* essential oil may also be achieved via a synergistic response of minor volatile compounds such as γ-terpinene, cuminyl alcohol, and β -pinene [50, 51]. A combination of terpenoid compounds has been reported to induce functional and physical decay of cell tissue of fungi, thereby causing cell membrane leakage [52]. Moreover, essential oils also have antimicrobial properties due to their disruption properties on the mycelial growth by reduction of microbial cellular metabolism [53, 54]. Uribe et al. [55] and Sikkema et al. [56] reported that their antifungal action of several monoterpenes was performed at the membrane level or embedded enzymes. Changing of the fatty acid composition of the cell membrane, alteration of permeability, and inhibition of respiration were suggested as possible mechanisms for antifungal propertied of monoterpenes [57, 58]. Marei & Abdelgaleil [59] also reported that the monoterpenes showed antifungal activity through the inhibition of pectin methyl esterase and cellulase enzymes. They were considered as the potent inhibitors of pectin methyl esterase modifying the degree of methylesterification of pectin, major component of fungal cell walls. Changing of pectin structure were resulted in cellular adhesion, plasticity, pH, and ionic contents of the cell wall and influenced fungi development, membrane integrity and permeability.

Evaluation of post-treatment grape berries quality indicates that *C*. *cyminum* essential oil having concentration of at least 400 μg/mL could delay the ripening process in grape berries by inhibiting *A*. *aculeatus* infection without affecting certain grape berries qualities after 10 days. The quality of fresh fruit depends on several factors including weight loss, firmness, total soluble solids, total phenolic content, and antioxidant activity. Serrano et al. [52] reported the efficacy of thymol and eugenol essential oils exposure on cherries and grapes without effects. Fennel and thyme oil were reported to reduce loss of grape weight [60] while cinnamon and eucalyptus essential oils do not affect weight loss in strawberries and tomato fruits [61]. In similar reports, *Ocimum basilicum* essential oil coated on Embul banana peel to control crown rot and anthracnose did not have any significant effect on the total soluble solid [62]. Ulukanli & Oz [63] reported that there are no significant differences in pH value in strawberries treated with myrtle essential oil. In addition, Wang et al. [64] found that thymol, eugenol, and menthol from essential oils enhanced phenolic and antioxidant activity in strawberries.

## 5. Conclusion

This investigation suggests *C*. *cyminum* essential oil as an alternative biological fungicide to control bunch rot diseases caused by *A*. *aculeatus* on postharvest grape berries. Considering all evaluated parameters on grape quality within the scope of this study, *C*. *cyminum* essential oil treatment was shown to delay grape ripening. Total phenolic content and antioxidant activity assay also showed that postharvest grape berries treated with *C*. *cyminum* essential oil could be stored while maintaining their quality for up to ten days. However, further studies are required before applying *C*. *cyminum* essential oil treatments as a biocontrol agent to increase the postharvest period of grapes.

## Supporting information

S1 Fig GC-MS chromatogram of *C*. *cyminum* essential oil. (TIF)

S1 File (DOCX)
