# Supplementary figures and images for: In vitro and in vivo antifungal activity of Cuminum cyminum essential oil against Aspergillus aculeatus causing bunch rot of postharvest grapes
Source: PLoS One. 2020 Nov 24;15(11):e0242862. doi: 10.1371/journal.pone.0242862 (PMC7685445; doi:10.1371/journal.pone.0242862)

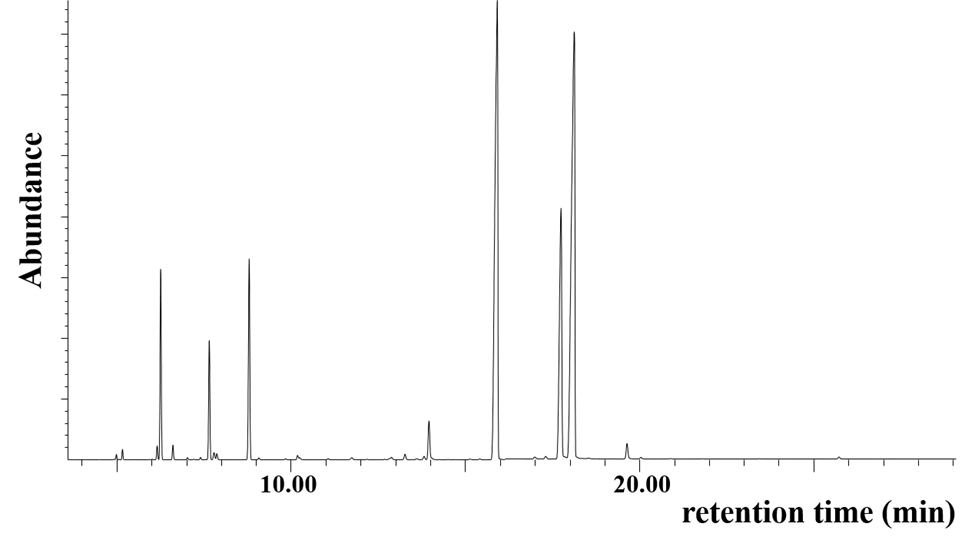

Supplement: S1 Fig — (TIF) [file pone.0242862.s001.tif]
