# Supplementary material for: In vitro and in vivo antifungal activity of Cuminum cyminum essential oil against Aspergillus aculeatus causing bunch rot of postharvest grapes
Source: PLoS One. 2020 Nov 24;15(11):e0242862. doi: 10.1371/journal.pone.0242862 (PMC7685445; doi:10.1371/journal.pone.0242862)
Supplement: S1 File — (DOCX) [file pone.0242862.s002.docx]

| **Figure/table** | | **a** | | **SD** | | **Statistical method** | **P value** | **# samples** |
| --- | --- | --- | --- | --- | --- | --- | --- | --- |
| **Figure 1:** The mycelial growth (cm) of *A. aculeatus* after transfer of discs where inhibition was complete (1000 µL/mL) during 14 days of incubation. | | | | | | | | |
| ***A. aculeatus* (treated)** |  | | |  | |  |  |  |
| Day 1 | 0.26 | | | 0.04 | | ANOVA and Duncan’s post-hoc test | P<0.05 | 15 |
| Day 2 | 0.41 | | | 0.01 | |  |  | 15 |
| Day 3 | 0.76 | | | 0.02 | |  |  | 15 |
| Day 4 | 1.08 | | | 0.02 | |  |  | 15 |
| Day 5 | 1.23 | | | 0.02 | |  |  | 15 |
| Day 6 | 1.48 | | | 0.02 | |  |  | 15 |
| Day 7 | 1.62 | | | 0.02 | |  |  | 15 |
| Day 8 | 1.79 | | | 0.04 | |  |  | 15 |
| Day 9 | 2.00 | | | 0.00 | |  |  | 15 |
| Day 10 | 2.05 | | | 0.08 | |  |  | 15 |
| Day 11 | 2.11 | | | 0.02 | |  |  | 15 |
| Day 12 | 2.23 | | | 0.02 | |  |  | 15 |
| Day 13 | 2.45 | | | 0.04 | |  |  | 15 |
| Day 14 | 2.50 | | | 0.02 | |  |  | 15 |
| ***A. aculeatus* (control)** |  | | |  | |  |  |  |
| Day 1 | 3.58 | | | 0.06 | | ANOVA and Duncan’s post-hoc test | P<0.05 | 15 |
| Day 2 | 5.62 | | | 0.03 | |  |  | 15 |
| Day 3 | 6.67 | | | 0.59 | |  |  | 15 |
| Day 4 | 7.93 | | | 0.25 | |  |  | 15 |
| Day 5 | 8.48 | | | 0.03 | |  |  | 15 |
| Day 6 | 8.60 | | | 0.00 | |  |  | 15 |
| Day 7 | 8.60 | | | 0.00 | |  |  | 15 |
| Day 8 | 8.60 | | | 0.00 | |  |  | 15 |
| Day 9 | 8.60 | | | 0.00 | |  |  | 15 |
| Day 10 | 8.60 | | | 0.00 | |  |  | 15 |
| Day 11 | 8.60 | | | 0.00 | |  |  | 15 |
| Day 12 | 8.60 | | | 0.00 | |  |  | 15 |
| Day 13 | 8.60 | | | 0.00 | |  |  | 15 |
| Day 14 | 8.60 | | | 0.00 | |  |  | 15 |
| **Figure 2:** Effect of different concentrations of *C. cyminum* essential oil (A), cumin aldehyde (B), and α-terpinen-7-al (C) on conidia germination of *A. aculeatus.* | | | | | | | | |
| **Fig. 1A** | |  | |  | |  |  |  |
| 0 µg/mL | | 100.00 | | 0.00 | | ANOVA and Duncan’s post-hoc test | P<0.05 | 15 |
| 200 µg/mL | | 18.80 | | 0.58 | |  |  | 15 |
| 400 µg/mL | | 16.40 | | 0.51 | |  |  | 15 |
| 600 µg/mL | | 12.00 | | 0.55 | |  |  | 15 |
| 800 µg/mL | | 4.80 | | 0.49 | |  |  | 15 |
| 1,000 µg/mL | | 1.00 | | 0.55 | |  |  | 15 |
| **Fig. 1B** | |  | |  | |  |  |  |
| 0 µg/mL | | 100.00 | | 0.00 | | ANOVA and Duncan’s post-hoc test | P<0.05 | 15 |
| 20 µg/mL | | 22.60 | | 0.40 | |  |  | 15 |
| 40 µg/mL | | 18.40 | | 0.66 | |  |  | 15 |
| 60 µg/mL | | 14.80 | | 0.37 | |  |  | 15 |
| 80 µg/mL | | 5.80 | | 0.51 | |  |  | 15 |
| 100 µg/mL | | 4.80 | | 0.93 | |  |  | 15 |
| **Fig. 1C** | |  | |  | |  |  |  |
| 0 µg/mL | | 100.00 | | 0.00 | | ANOVA and Duncan’s post-hoc test | P<0.05 | 15 |
| 20 µg/mL | | 25.60 | | 0.75 | |  |  | 15 |
| 40 µg/mL | | 20.80 | | 0.68 | |  |  | 15 |
| 60 µg/mL | | 17.20 | | 0.37 | |  |  | 15 |
| 80 µg/mL | | 14.60 | | 0.66 | |  |  | 15 |
| 100 µg/mL | | 7.40 | | 1.32 | |  |  | 15 |
| **Figure 5:** Incidence percentage of *A. aculeatus* in grape berries treated with different concentrations of *C. cyminum* essential oil (A), cumin aldehyde (B), and α-terpinen-7-al (C). | | | | | | | | |
| **Fig. 5A** | |  | |  | |  |  |  |
| 0 µg/mL | | 10.00 | | 0.50 | | ANOVA and Duncan’s post-hoc test | P<0.05 | 15 |
| 200 µg/mL | | 8.33 | | 0.32 | |  |  | 15 |
| 400 µg/mL | | 6.85 | | 0.19 | |  |  | 15 |
| 600 µg/mL | | 5.56 | | 0.32 | |  |  | 15 |
| 800 µg/mL | | 3.52 | | 0.19 | |  |  | 15 |
| 1,000 µg/mL | | 1.48 | | 0.19 | |  |  | 15 |
| **Fig. 5B** | |  | |  | |  |  |  |
| 0 µg/mL | | 9.70 | | 0.50 | | ANOVA and Duncan’s post-hoc test | P<0.05 | 15 |
| 20 µg/mL | | 9.63 | | 0.19 | |  |  | 15 |
| 40 µg/mL | | 7.78 | | 0.32 | |  |  | 15 |
| 60 µg/mL | | 6.30 | | 0.19 | |  |  | 15 |
| 80 µg/mL | | 4.44 | | 0.32 | |  |  | 15 |
| 100 µg/mL | | 2.04 | | 0.19 | |  |  | 15 |
| **Fig. 5C** | |  | |  | |  |  |  |
| 0 µg/mL | | 10.00 | | 0.30 | | ANOVA and Duncan’s post-hoc test | P<0.05 | 15 |
| 20 µg/mL | | 9.81 | | 0.32 | |  |  | 15 |
| 40 µg/mL | | 8.52 | | 0.37 | |  |  | 15 |
| 60 µg/mL | | 7.41 | | 0.19 | |  |  | 15 |
| 80 µg/mL | | 6.48 | | 0.19 | |  |  | 15 |
| 100 µg/mL | | 6.48 | | 0.19 | |  |  | 15 |
| **Figure 5:** Severity percentage of *A. aculeatus* in grape berries treated with different concentrations of *C. cyminum* essential oil (A), cumin aldehyde (B), and α-terpinen-7-al (C). | | | | | | | | |
| **Fig. 5A** | |  | |  | |  |  |  |
| 0 µg/mL | | 15.00 | | 0.20 | | ANOVA and Duncan’s post-hoc test | P<0.05 | 15 |
| 200 µg/mL | | 10.88 | | 0.23 | |  |  | 15 |
| 400 µg/mL | | 7.59 | | 0.19 | |  |  | 15 |
| 600 µg/mL | | 4.03 | | 0.14 | |  |  | 15 |
| 800 µg/mL | | 1.76 | | 0.09 | |  |  | 15 |
| 1,000 µg/mL | | 0.69 | | 0.14 | |  |  | 15 |
| **Fig. 5B** | |  | |  | |  |  |  |
| 0 µg/mL | | 15.00 | | 0.20 | | ANOVA and Duncan’s post-hoc test | P<0.05 | 15 |
| 20 µg/mL | | 12.27 | | 0.23 | |  |  | 15 |
| 40 µg/mL | | 7.96 | | 0.19 | |  |  | 15 |
| 60 µg/mL | | 4.58 | | 0.24 | |  |  | 15 |
| 80 µg/mL | | 2.22 | | 0.16 | |  |  | 15 |
| 100 µg/mL | | 1.11 | | 0.14 | |  |  | 15 |
| **Fig. 5C** | |  | |  | |  |  |  |
| 0 µg/mL | | 15.00 | | 0.30 | | ANOVA and Duncan’s post-hoc test | P<0.05 | 15 |
| 20 µg/mL | | 14.72 | | 0.28 | |  |  | 15 |
| 40 µg/mL | | 11.94 | | 0.48 | |  |  | 15 |
| 60 µg/mL | | 10.56 | | 0.28 | |  |  | 15 |
| 80 µg/mL | | 8.56 | | 0.40 | |  |  | 15 |
| 100 µg/mL | | 8.10 | | 0.23 | |  |  | 15 |
| **Figure 5:** Incidence percentage of *A. aculeatus* in grape berries treated with different concentrations of C. *cyminum* essential oil (D), cumin aldehyde (E), and α-terpinen-7-al (F) after 20 days | | | | | | | | |
| **Fig. 5D** | |  | | |  |  | |  |
| 0 µg/mL | | 35.00 | | | 0.50 | ANOVA and Duncan’s post-hoc test | P<0.05 | 15 |
| 200 µg/mL | | 31.76 | | | 0.65 |  |  | 15 |
| 400 µg/mL | | 22.69 | | | 0.65 |  |  | 15 |
| 600 µg/mL | | 12.31 | | | 0.65 |  |  | 15 |
| 800 µg/mL | | 6.48 | | | 0.65 |  |  | 15 |
| 1000 µg/mL | | 2.59 | | | 0.65 |  |  | 15 |
| **Fig. 5E** | |  | | |  |  |  |  |
| 0 µg/mL | | 35.00 | | 0.50 | | ANOVA and Duncan’s post-hoc test | P<0.05 | 15 |
| 20 µg/mL | | 29.17 | | 0.83 | |  |  | 15 |
| 40 µg/mL | | 23.33 | | 0.83 | |  |  | 15 |
| 60 µg/mL | | 13.33 | | 0.83 | |  |  | 15 |
| 80 µg/mL | | 6.67 | | 0.83 | |  |  | 15 |
| 100 µg/mL | | 3.33 | | 0.83 | |  |  | 15 |
| **Fig. 5F** | |  | |  | |  |  |  |
| 0 µg/mL | | 35.00 | | 0.50 | | ANOVA and Duncan’s post-hoc test | P<0.05 | 15 |
| 20 µg/mL | | 24.54 | | 0.32 | |  |  | 15 |
| 40 µg/mL | | 21.76 | | 0.46 | |  |  | 15 |
| 60 µg/mL | | 20.37 | | 0.93 | |  |  | 15 |
| 80 µg/mL | | 19.91 | | 0.46 | |  |  | 15 |
| 100 µg/mL | | 18.06 | | 0.80 | |  |  | 15 |
| **Figure 5:** Severity percentage of *A. aculeatus* in grape berries treated with different concentrations of *C.* *cyminum* essential oil (D), cumin aldehyde (E), and α-terpinen-7-al (F) after 20 days. | | | | | | | | |
| **Fig. 5D** | |  | |  | |  |  |  |
| 0 µg/mL | | 45.00 | | 0.20 | | ANOVA and Duncan’s post-hoc test | P<0.05 | 15 |
| 200 µg/mL | | 39.81 | | 0.93 | |  |  | 15 |
| 400 µg/mL | | 28.70 | | 0.93 | |  |  | 15 |
| 600 µg/mL | | 18.52 | | 0.93 | |  |  | 15 |
| 800 µg/mL | | 9.63 | | 0.74 | |  |  | 15 |
| 1000 µg/mL | | 3.70 | | 0.93 | |  |  | 15 |
| **Fig. 4E** | |  | |  | |  | |  |
| 0 µg/mL | | 45.00 | | 0.20 | | ANOVA and Duncan’s post-hoc test | P<0.05 | 15 |
| 20 µg/mL | | 37.16 | | 0.86 | |  |  | 15 |
| 40 µg/mL | | 29.38 | | 0.86 | |  |  | 15 |
| 60 µg/mL | | 21.48 | | 0.74 | |  |  | 15 |
| 80 µg/mL | | 12.59 | | 0.74 | |  |  | 15 |
| 100 µg/mL | | 5.56 | | 1.07 | |  |  | 15 |
| **Fig. 4F** | |  | |  | |  |  |  |
| 0 µg/mL | | 45.00 | | 0.20 | | ANOVA and Duncan’s post-hoc test | P<0.05 | 15 |
| 20 µg/mL | | 39.75 | | 0.86 | |  |  | 15 |
| 40 µg/mL | | 35.43 | | 0.86 | |  |  | 15 |
| 60 µg/mL | | 33.70 | | 1.50 | |  |  | 15 |
| 80 µg/mL | | 29.63 | | 0.74 | |  |  | 15 |
| 100 µg/mL | | 25.93 | | 0.74 | |  |  | 15 |
| **Table 1**: Antifungal activity of *C. cyminum, Z. bungeanum* Maxim., *C. sativum, Z. montanum* (J. Koenig) Link ex A. Dietr., and *Z. rhetsa* essential oils at different concentrations against *A. aculeatus* using contact phase assay. | | | | | | | | |
| ***C. cyminum*** | |  | |  | |  |  |  |
| **Day 1** | |  | |  | |  |  |  |
| 200 µg/mL | | 78.27 | | 0.28 | | ANOVA and Duncan’s post-hoc test | P<0.05 | 15 |
| 400 µg/mL | | 82.06 | | 0.81 | |  |  | 15 |
| 600 µg/mL | | 85.04 | | 0.23 | |  |  | 15 |
| 800 µg/mL | | 91.09 | | 0.34 | |  |  | 15 |
| 1,000 µg/mL | | 95.08 | | 0.12 | |  |  | 15 |
| **Day 2** | |  | |  | |  |  |  |
| 200 µg/mL | | 76.44 | | 1.09 | | ANOVA and Duncan’s post- hoc test | P<0.05 | 15 |
| 400 µg/mL | | 80.79 | | 0.30 | |  |  | 15 |
| 600 µg/mL | | 84.13 | | 0.53 | |  |  | 15 |
| 800 µg/mL | | 90.23 | | 0.25 | |  |  | 15 |
| 1,000 µg/mL | | 92.25 | | 0.38 | |  |  | 15 |
| **Day 3** | |  | |  | |  |  |  |
| 200 µg/mL | | 76.37 | | 0.33 | | ANOVA and Duncan’s post- hoc test | P<0.05 | 15 |
| 400 µg/mL | | 80.05 | | 0.50 | |  |  | 15 |
| 600 µg/mL | | 83.75 | | 0.93 | |  |  | 15 |
| 800 µg/mL | | 87.05 | | 0.56 | |  |  | 15 |
| 1,000 µg/mL | | 89.29 | | 0.51 | |  |  | 15 |
| **Day 4** | |  | |  | |  |  |  |
| 200 µg/mL | | 72.66 | | 0.58 | | ANOVA and Duncan’s post- hoc test | P<0.05 | 15 |
| 400 µg/mL | | 79.27 | | 0.60 | |  |  | 15 |
| 600 µg/mL | | 83.43 | | 0.28 | |  |  | 15 |
| 800 µg/mL | | 86.09 | | 1.12 | |  |  | 15 |
| 1,000 µg/mL | | 87.64 | | 0.52 | |  |  | 15 |
| **Day 5** | |  | |  | |  |  |  |
| 200 µg/mL | | 72.64 | | 0.98 | | ANOVA and Duncan’s post- hoc test | P<0.05 | 15 |
| 400 µg/mL | | 79.08 | | 0.27 | |  |  | 15 |
| 600 µg/mL | | 82.43 | | 0.47 | |  |  | 15 |
| 800 µg/mL | | 85.49 | | 0.43 | |  |  | 15 |
| 1,000 µg/mL | | 87.20 | | 0.85 | |  |  | 15 |
| **Day 6** | |  | |  | |  |  |  |
| 200 µg/mL | | 70.41 | | 0.83 | | ANOVA and Duncan’s post- hoc test | P<0.05 | 15 |
| 400 µg/mL | | 76.19 | | 1.13 | |  |  | 15 |
| 600 µg/mL | | 79.87 | | 0.90 | |  |  | 15 |
| 800 µg/mL | | 82.55 | | 0.90 | |  |  | 15 |
| 1,000 µg/mL | | 86.04 | | 0.57 | |  |  | 15 |
| **Day 7** | |  | |  | |  |  |  |
| 200 µg/mL | | 69.69 | | 1.68 | | ANOVA and Duncan’s post-hoc test | P<0.05 | 15 |
| 400 µg/mL | | 75.76 | | 0.76 | |  |  | 15 |
| 600 µg/mL | | 76.13 | | 1.49 | |  |  | 15 |
| 800 µg/mL | | 81.48 | | 0.66 | |  |  | 15 |
| 1,000 µg/mL | | 82.13 | | 1.49 | |  |  | 15 |
| ***Z. bungeanum*** | |  | |  | |  |  |  |
| **Day 1** | |  | |  | |  |  |  |
| 200 µg/mL | | 38.52 | | 2.00 | | ANOVA and Duncan’s post- hoc test | P<0.05 | 15 |
| 400 µg/mL | | 39.26 | | 0.98 | |  |  | 15 |
| 600 µg/mL | | 44.97 | | 1.81 | |  |  | 15 |
| 800 µg/mL | | 47.72 | | 1.79 | |  |  | 15 |
| 1,000 µg/mL | | 50.39 | | 1.98 | |  |  | 15 |
| **Day 2** | |  | |  | |  |  |  |
| 200 µg/mL | | 34.09 | | 0.83 | | ANOVA and Duncan’s post-hoc test | P<0.05 | 15 |
| 400 µg/mL | | 36.94 | | 1.29 | |  |  | 15 |
| 600 µg/mL | | 40.65 | | 1.42 | |  |  | 15 |
| 800 µg/mL | | 44.69 | | 1.31 | |  |  | 15 |
| 1,000 µg/mL | | 47.73 | | 1.42 | |  |  | 15 |
| **Day 3** | |  | |  | |  |  |  |
| 200 µg/mL | | 31.47 | | 1.26 | | ANOVA and Duncan’s post- hoc test | P<0.05 | 15 |
| 400 µg/mL | | 33.87 | | 1.42 | |  |  | 15 |
| 600 µg/mL | | 39.29 | | 1.35 | |  |  | 15 |
| 800 µg/mL | | 43.03 | | 1.05 | |  |  | 15 |
| 1,000 µg/mL | | 45.20 | | 1.66 | |  |  | 15 |
| **Day 4** | |  | |  | |  |  |  |
| 200 µg/mL | | 27.49 | | 0.00 | | ANOVA and Duncan’s post- hoc test | P<0.05 | 15 |
| 400 µg/mL | | 31.03 | | 1.13 | |  |  | 15 |
| 600 µg/mL | | 37.83 | | 1.77 | |  |  | 15 |
| 800 µg/mL | | 41.75 | | 1.61 | |  |  | 15 |
| 1,000 µg/mL | | 43.66 | | 0.95 | |  |  | 15 |
| **Day 5** | |  | |  | |  |  |  |
| 200 µg/mL | | 25.57 | | 1.15 | | ANOVA and Duncan’s post- hoc test | P<0.05 | 15 |
| 400 µg/mL | | 29.57 | | 1.00 | |  |  | 15 |
| 600 µg/mL | | 36.31 | | 0.88 | |  |  | 15 |
| 800 µg/mL | | 39.10 | | 1.69 | |  |  | 15 |
| 1,000 µg/mL | | 41.82 | | 1.37 | |  |  | 15 |
| **Day 6** | |  | |  | |  |  |  |
| 200 µg/mL | | 24.21 | | 0.00 | | ANOVA and Duncan’s post-hoc test | P<0.05 | 15 |
| 400 µg/mL | | 28.91 | | 0.96 | |  |  | 15 |
| 600 µg/mL | | 35.05 | | 1.46 | |  |  | 15 |
| 800 µg/mL | | 37.25 | | 0.82 | |  |  | 15 |
| 1,000 µg/mL | | 41.22 | | 0.52 | |  |  | 15 |
| **Day 7** | |  | |  | |  |  |  |
| 200 µg/mL | | 22.49 | | 1.01 | | ANOVA and Duncan’s post-hoc test | P<0.05 | 15 |
| 400 µg/mL | | 26.91 | | 0.94 | |  |  | 15 |
| 600 µg/mL | | 33.46 | | 0.84 | |  |  | 15 |
| 800 µg/mL | | 35.05 | | 1.42 | |  |  | 15 |
| 1,000 µg/mL | | 39.10 | | 0.53 | |  |  | 15 |
| ***C. sativum*** | |  | |  | |  |  |  |
| **Day 1** | |  | |  | |  |  |  |
| 200 µg/mL | | 41.27 | | 0.68 | | ANOVA and Duncan’s post- hoc test | P<0.05 | 15 |
| 400 µg/mL | | 45.85 | | 0.54 | |  |  | 15 |
| 600 µg/mL | | 47.84 | | 0.64 | |  |  | 15 |
| 800 µg/mL | | 50.98 | | 0.88 | |  |  | 15 |
| 1,000 µg/mL | | 53.38 | | 0.30 | |  |  | 15 |
| **Day 2** | |  | |  | |  |  |  |
| 200 µg/mL | | 29.77 | | 0.40 | | ANOVA and Duncan’s post- hoc test | P<0.05 | 15 |
| 400 µg/mL | | 34.22 | | 0.39 | |  |  | 15 |
| 600 µg/mL | | 46.64 | | 1.07 | |  |  | 15 |
| 800 µg/mL | | 49.06 | | 0.74 | |  |  | 15 |
| 1,000 µg/mL | | 51.00 | | 0.47 | |  |  | 15 |
| **Day 3** | |  | |  | |  |  |  |
| 200 µg/mL | | 27.18 | | 1.08 | | ANOVA and Duncan’s post-hoc test | P<0.05 | 15 |
| 400 µg/mL | | 30.62 | | 0.91 | |  |  | 15 |
| 600 µg/mL | | 43.60 | | 0.51 | |  |  | 15 |
| 800 µg/mL | | 47.44 | | 0.37 | |  |  | 15 |
| 1,000 µg/mL | | 50.06 | | 0.37 | |  |  | 15 |
| **Day 4** | |  | |  | |  |  |  |
| 200 µg/mL | | 26.94 | | 0.54 | | ANOVA and Duncan’s post- hoc test | P<0.05 | 15 |
| 400 µg/mL | | 30.54 | | 1.45 | |  |  | 15 |
| 600 µg/mL | | 43.25 | | 1.42 | |  |  | 15 |
| 800 µg/mL | | 45.13 | | 0.38 | |  |  | 15 |
| 1,000 µg/mL | | 48.74 | | 0.58 | |  |  | 15 |
| **Day 5** | |  | |  | |  |  |  |
| 200 µg/mL | | 23.62 | | 0.55 | | ANOVA and Duncan’s post-hoc test | P<0.05 | 15 |
| 400 µg/mL | | 22.79 | | 0.92 | |  |  | 15 |
| 600 µg/mL | | 31.52 | | 0.90 | |  |  | 15 |
| 800 µg/mL | | 31.19 | | 1.34 | |  |  | 15 |
| 1,000 µg/mL | | 47.65 | | 0.98 | |  |  | 15 |
| **Day 6** | |  | |  | |  |  |  |
| 200 µg/mL | | 21.69 | | 0.98 | | ANOVA and Duncan’s post-hoc test | P<0.05 | 15 |
| 400 µg/mL | | 21.94 | | 0.93 | |  |  | 15 |
| 600 µg/mL | | 29.40 | | 0.53 | |  |  | 15 |
| 800 µg/mL | | 31.22 | | 0.80 | |  |  | 15 |
| 1,000 µg/mL | | 36.37 | | 0.87 | |  |  | 15 |
| **Day 7** | |  | |  | |  |  |  |
| 200 µg/mL | | 21.39 | | 1.20 | | ANOVA and Duncan’s post- hoc test | P<0.05 | 15 |
| 400 µg/mL | | 21.11 | | 1.64 | |  |  | 15 |
| 600 µg/mL | | 22.50 | | 0.67 | |  |  | 15 |
| 800 µg/mL | | 28.52 | | 1.27 | |  |  | 15 |
| 1,000 µg/mL | | 36.00 | | 0.75 | |  |  | 15 |
| ***Z. montanum*** | |  | |  | |  |  |  |
| **Day 1** | |  | |  | |  |  |  |
| 200 µg/mL | | 40.13 | | 0.56 | | ANOVA and Duncan’s post- hoc test | P<0.05 | 15 |
| 400 µg/mL | | 43.66 | | 0.95 | |  |  | 15 |
| 600 µg/mL | | 46.46 | | 0.92 | |  |  | 15 |
| 800 µg/mL | | 50.97 | | 1.54 | |  |  | 15 |
| 1,000 µg/mL | | 51.86 | | 0.88 | |  |  | 15 |
| **Day 2** | |  | |  | |  |  |  |
| 200 µg/mL | | 39.29 | | 1.32 | | ANOVA and Duncan’s post- hoc test | P<0.05 | 15 |
| 400 µg/mL | | 42.00 | | 2.06 | |  |  | 15 |
| 600 µg/mL | | 44.24 | | 1.33 | |  |  | 15 |
| 800 µg/mL | | 49.06 | | 0.97 | |  |  | 15 |
| 1,000 µg/mL | | 51.51 | | 0.59 | |  |  | 15 |
| **Day 3** | |  | |  | |  |  |  |
| 200 µg/mL | | 37.25 | | 0.54 | | ANOVA and Duncan’s post-hoc test | P<0.05 | 15 |
| 400 µg/mL | | 41.91 | | 1.70 | |  |  | 15 |
| 600 µg/mL | | 43.31 | | 0.89 | |  |  | 15 |
| 800 µg/mL | | 45.56 | | 1.31 | |  |  | 15 |
| 1,000 µg/mL | | 50.72 | | 0.48 | |  |  | 15 |
| **Day 4** | |  | |  | |  |  |  |
| 200 µg/mL | | 26.47 | | 1.64 | | ANOVA and Duncan’s post- hoc test | P<0.05 | 15 |
| 400 µg/mL | | 28.79 | | 0.92 | |  |  | 15 |
| 600 µg/mL | | 43.00 | | 1.82 | |  |  | 15 |
| 800 µg/mL | | 44.48 | | 2.18 | |  |  | 15 |
| 1,000 µg/mL | | 48.78 | | 0.49 | |  |  | 15 |
| **Day 5** | |  | |  | |  |  |  |
| 200 µg/mL | | 24.74 | | 1.09 | | ANOVA and Duncan’s post- hoc test | P<0.05 | 15 |
| 400 µg/mL | | 25.05 | | 0.95 | |  |  | 15 |
| 600 µg/mL | | 30.31 | | 1.39 | |  |  | 15 |
| 800 µg/mL | | 42.71 | | 1.03 | |  |  | 15 |
| 1,000 µg/mL | | 47.52 | | 1.12 | |  |  | 15 |
| **Day 6** | |  | |  | |  |  |  |
| 200 µg/mL | | 23.65 | | 0.66 | | ANOVA and Duncan’s post- hoc test | P<0.05 | 15 |
| 400 µg/mL | | 24.03 | | 1.32 | |  |  | 15 |
| 600 µg/mL | | 28.18 | | 0.53 | |  |  | 15 |
| 800 µg/mL | | 40.62 | | 0.91 | |  |  | 15 |
| 1,000 µg/mL | | 45.64 | | 0.50 | |  |  | 15 |
| **Day 7** | |  | |  | |  |  |  |
| 200 µg/mL | | 23.14 | | 0.96 | | ANOVA and Duncan’s post- hoc test | P<0.05 | 15 |
| 400 µg/mL | | 23.78 | | 0.55 | |  |  | 15 |
| 600 µg/mL | | 25.16 | | 1.74 | |  |  | 15 |
| 800 µg/mL | | 40.55 | | 1.13 | |  |  | 15 |
| 1,000 µg/mL | | 45.12 | | 1.29 | |  |  | 15 |
| ***Z. rhetsa*** | |  | |  | |  |  |  |
| **Day 1** | |  | |  | |  |  |  |
| 200 µg/mL | | 46.16 | | 0.54 | | ANOVA and Duncan’s post- hoc test | P<0.05 | 15 |
| 400 µg/mL | | 48.75 | | 0.64 | |  |  | 15 |
| 600 µg/mL | | 49.19 | | 1.27 | |  |  | 15 |
| 800 µg/mL | | 52.29 | | 1.85 | |  |  | 15 |
| 1,000 µg/mL | | 54.16 | | 0.98 | |  |  | 15 |
| **Day 2** | |  | |  | |  |  |  |
| 200 µg/mL | | 45.56 | | 2.16 | | ANOVA and Duncan’s post- hoc test | P<0.05 | 15 |
| 400 µg/mL | | 46.63 | | 2.13 | |  |  | 15 |
| 600 µg/mL | | 48.75 | | 1.82 | |  |  | 15 |
| 800 µg/mL | | 51.51 | | 0.29 | |  |  | 15 |
| 1,000 µg/mL | | 53.52 | | 0.58 | |  |  | 15 |
| **Day 3** | |  | |  | |  |  |  |
| 200 µg/mL | | 33.36 | | 0.77 | | ANOVA and Duncan’s post- hoc test | P<0.05 | 15 |
| 400 µg/mL | | 44.24 | | 1.33 | |  |  | 15 |
| 600 µg/mL | | 45.79 | | 0.38 | |  |  | 15 |
| 800 µg/mL | | 48.07 | | 0.49 | |  |  | 15 |
| 1,000 µg/mL | | 51.10 | | 0.72 | |  |  | 15 |
| **Day 4** | |  | |  | |  |  |  |
| 200 µg/mL | | 32.56 | | 0.54 | | ANOVA and Duncan’s post- hoc test | P<0.05 | 15 |
| 400 µg/mL | | 31.29 | | 1.13 | |  |  | 15 |
| 600 µg/mL | | 45.50 | | 0.61 | |  |  | 15 |
| 800 µg/mL | | 46.86 | | 2.23 | |  |  | 15 |
| 1,000 µg/mL | | 50.45 | | 0.48 | |  |  | 15 |
| **Day 5** | |  | |  | |  |  |  |
| 200 µg/mL | | 14.74 | | 0.55 | | ANOVA and Duncan’s post- hoc test | P<0.05 | 15 |
| 400 µg/mL | | 27.40 | | 0.93 | |  |  | 15 |
| 600 µg/mL | | 40.16 | | 0.64 | |  |  | 15 |
| 800 µg/mL | | 44.63 | | 0.51 | |  |  | 15 |
| 1,000 µg/mL | | 47.36 | | 1.31 | |  |  | 15 |
| **Day 6** | |  | |  | |  |  |  |
| 200 µg/mL | | 12.50 | | 0.56 | | ANOVA and Duncan’s post- hoc test | P<0.05 | 15 |
| 400 µg/mL | | 24.78 | | 1.73 | |  |  | 15 |
| 600 µg/mL | | 26.94 | | 0.66 | |  |  | 15 |
| 800 µg/mL | | 31.97 | | 0.52 | |  |  | 15 |
| 1,000 µg/mL | | 44.62 | | 1.60 | |  |  | 15 |
| **Day 7** | |  | |  | |  |  |  |
| 200 µg/mL | | 12.11 | | 1.64 | | ANOVA and Duncan’s post- hoc test | P<0.05 | 15 |
| 400 µg/mL | | 24.58 | | 0.55 | |  |  | 15 |
| 600 µg/mL | | 26.29 | | 1.72 | |  |  | 15 |
| 800 µg/mL | | 28.52 | | 1.27 | |  |  | 15 |
| 1,000 µg/mL | | 42.42 | | 0.89 | |  |  | 15 |
| **Table 3:** Antifungal activity of cumin aldehyde and α-terpinen-7-al at different concentrations against *A*. *aculeatus* using contact phase assay. | | | | | | | | |
| **Cumin aldehyde** | | |  |  | |  |  |  |
| **Day 1** | |  | |  | |  |  |  |
| 20 µg/mL | | 72.04 | | 0.85 | | ANOVA and Duncan’s post- hoc test | P<0.05 | 15 |
| 40 µg/mL | | 77.13 | | 0.76 | |  |  | 15 |
| 60 µg/mL | | 80.11 | | 0.71 | |  |  | 15 |
| 80 µg/mL | | 82.10 | | 0.67 | |  |  | 15 |
| 100 µg/mL | | 85.07 | | 1.07 | |  |  | 15 |
| **Day 2** | |  | |  | |  |  |  |
| 20 µg/mL | | 69.55 | | 1.52 | | ANOVA and Duncan’s post- hoc test | P<0.05 | 15 |
| 40 µg/mL | | 71.06 | | 0.00 | |  |  | 15 |
| 60 µg/mL | | 74.42 | | 0.80 | |  |  | 15 |
| 80 µg/mL | | 78.86 | | 0.74 | |  |  | 15 |
| 100 µg/mL | | 81.32 | | 0.69 | |  |  | 15 |
| **Day 3** | |  | |  | |  |  |  |
| 20 µg/mL | | 62.61 | | 0.98 | | ANOVA and Duncan’s post- hoc test | P<0.05 | 15 |
| 40 µg/mL | | 70.55 | | 1.71 | |  |  | 15 |
| 60 µg/mL | | 72.04 | | 0.85 | |  |  | 15 |
| 80 µg/mL | | 74.88 | | 0.80 | |  |  | 15 |
| 100 µg/mL | | 78.43 | | 0.74 | |  |  | 15 |
| **Day 4** | |  | |  | |  |  |  |
| 20 µg/mL | | 60.90 | | 1.00 | | ANOVA and Duncan’s post- hoc test | P<0.05 | 15 |
| 40 µg/mL | | 64.81 | | 1.64 | |  |  | 15 |
| 60 µg/mL | | 69.55 | | 1.52 | |  |  | 15 |
| 80 µg/mL | | 71.55 | | 0.85 | |  |  | 15 |
| 100 µg/mL | | 75.33 | | 1.37 | |  |  | 15 |
| **Day 5** | |  | |  | |  |  |  |
| 20 µg/mL | | 58.57 | | 1.02 | | ANOVA and Duncan’s post- hoc test | P<0.05 | 15 |
| 40 µg/mL | | 61.46 | | 1.71 | |  |  | 15 |
| 60 µg/mL | | 66.44 | | 0.00 | |  |  | 15 |
| 80 µg/mL | | 68.53 | | 0.89 | |  |  | 15 |
| 100 µg/mL | | 72.52 | | 1.45 | |  |  | 15 |
| **Day 6** | |  | |  | |  |  |  |
| 20 µg/mL | | 56.77 | | 1.04 | | ANOVA and Duncan’s post- hoc test | P<0.05 | 15 |
| 40 µg/mL | | 59.16 | | 1.02 | |  |  | 15 |
| 60 µg/mL | | 63.16 | | 1.67 | |  |  | 15 |
| 80 µg/mL | | 64.27 | | 0.96 | |  |  | 15 |
| 100 µg/mL | | 68.53 | | 0.89 | |  |  | 15 |
| **Day 7** | |  | |  | |  |  |  |
| 20 µg/mL | | 54.94 | | 1.07 | | ANOVA and Duncan’s post- hoc test | P<0.05 | 15 |
| 40 µg/mL | | 56.16 | | 1.83 | |  |  | 15 |
| 60 µg/mL | | 60.90 | | 1.00 | |  |  | 15 |
| 80 µg/mL | | 62.04 | | 0.98 | |  |  | 15 |
| 100 µg/mL | | 65.36 | | 0.93 | |  |  | 15 |
| **α-terpinen-7-al** | |  | |  | |  |  |  |
| **Day 1** | |  | |  | |  |  |  |
| 20 µg/mL | | 54.94 | | 1.07 | | ANOVA and Duncan’s post- hoc test | P<0.05 | 15 |
| 40 µg/mL | | 59.16 | | 1.02 | |  |  | 15 |
| 60 µg/mL | | 62.61 | | 0.98 | |  |  | 15 |
| 80 µg/mL | | 66.97 | | 0.91 | |  |  | 15 |
| 100 µg/mL | | 71.55 | | 0.85 | |  |  | 15 |
| **Day 2** | |  | |  | |  |  |  |
| 20 µg/mL | | 51.16 | | 1.11 | | ANOVA and Duncan’s post- hoc test | P<0.05 | 15 |
| 40 µg/mL | | 55.55 | | 1.07 | |  |  | 15 |
| 60 µg/mL | | 60.32 | | 1.00 | |  |  | 15 |
| 80 µg/mL | | 63.72 | | 0.96 | |  |  | 15 |
| 100 µg/mL | | 67.49 | | 0.91 | |  |  | 15 |
| **Day 3** | |  | |  | |  |  |  |
| 20 µg/mL | | 48.55 | | 1.98 | | ANOVA and Duncan’s post- hoc test | P<0.05 | 15 |
| 40 µg/mL | | 51.80 | | 1.11 | |  |  | 15 |
| 60 µg/mL | | 57.37 | | 1.04 | |  |  | 15 |
| 80 µg/mL | | 59.16 | | 1.02 | |  |  | 15 |
| 100 µg/mL | | 63.72 | | 0.96 | |  |  | 15 |
| **Day 4** | |  | |  | |  |  |  |
| 20 µg/mL | | 45.88 | | 1.18 | | ANOVA and Duncan’s post- hoc test | P<0.05 | 15 |
| 40 µg/mL | | 49.21 | | 1.13 | |  |  | 15 |
| 60 µg/mL | | 53.70 | | 1.09 | |  |  | 15 |
| 80 µg/mL | | 56.16 | | 1.83 | |  |  | 15 |
| 100 µg/mL | | 59.16 | | 1.02 | |  |  | 15 |
| **Day 5** | |  | |  | |  |  |  |
| 20 µg/mL | | 41.04 | | 1.22 | | ANOVA and Duncan’s post- hoc test | P<0.05 | 15 |
| 40 µg/mL | | 47.23 | | 1.15 | |  |  | 15 |
| 60 µg/mL | | 50.52 | | 0.00 | |  |  | 15 |
| 80 µg/mL | | 53.07 | | 1.09 | |  |  | 15 |
| 100 µg/mL | | 55.55 | | 1.07 | |  |  | 15 |
| **Day 6** | |  | |  | |  |  |  |
| 20 µg/mL | | 38.18 | | 2.17 | | ANOVA and Duncan’s post- hoc test | P<0.05 | 15 |
| 40 µg/mL | | 43.14 | | 1.20 | |  |  | 15 |
| 60 µg/mL | | 47.23 | | 1.15 | |  |  | 15 |
| 80 µg/mL | | 49.21 | | 1.13 | |  |  | 15 |
| 100 µg/mL | | 51.80 | | 1.11 | |  |  | 15 |
| **Day 7** | |  | |  | |  |  |  |
| 20 µg/mL | | 33.01 | | 2.59 | | ANOVA and Duncan’s post- hoc test | P<0.05 | 15 |
| 40 µg/mL | | 39.62 | | 1.24 | |  |  | 15 |
| 60 µg/mL | | 43.82 | | 2.37 | |  |  | 15 |
| 80 µg/mL | | 45.20 | | 1.18 | |  |  | 15 |
| 100 µg/mL | | 47.89 | | 1.15 | |  |  | 15 |
| **Table 4:** Quality of grape berries obtained from all treatments | | | | | | | | |
| **CC** | |  | |  | |  |  |  |
| % Weight loss | | 2.08 | | 0.01 | | ANOVA and Duncan’s post-hoc test | P<0.05 | 15 |
| Firmness (N) | | 0.45 | | 0.01 | |  |  | 15 |
| Total phenolic content | | 7.07 | | 0.01 | |  |  | 15 |
| Antioxidant activity | | 29.83 | | 0.07 | |  |  | 15 |
| pH | | 3.41 | | 0.00 | |  |  | 15 |
| TSS | | 19.66 | | 0.03 | |  |  | 15 |
| **PC** | |  | |  | |  |  |  |
| % Weight loss | | 3.32 | | 0.02 | | ANOVA and Duncan’s post- hoc test | P<0.05 | 15 |
| Firmness (N) | | 0.31 | | 0.01 | |  |  | 15 |
| Total phenolic content | | 3.92 | | 0.01 | |  |  | 15 |
| Antioxidant activity | | 18.63 | | 0.07 | |  |  | 15 |
| pH | | 3.24 | | 0.01 | |  |  | 15 |
| TSS | | 17.70 | | 0.19 | |  |  | 15 |
| ***C. cyminum* NC** | |  | |  | |  |  |  |
| % Weight loss | | 2.11 | | 0.01 | | ANOVA and Duncan’s post- hoc test | P<0.05 | 15 |
| Firmness (N) | | 0.41 | | 0.01 | |  |  | 15 |
| Total phenolic content | | 7.33 | | 0.03 | |  |  | 15 |
| Antioxidant activity | | 27.42 | | 0.23 | |  |  | 15 |
| pH | | 3.42 | | 0.01 | |  |  | 15 |
| TSS | | 18.63 | | 0.06 | |  |  | 15 |
| **Cumin aldehyde NC** | |  | |  | |  |  |  |
| % Weight loss | | 2.08 | | 0.01 | | ANOVA and Duncan’s post- hoc test | P<0.05 | 15 |
| Firmness (N) | | 0.42 | | 0.01 | |  |  | 15 |
| Total phenolic content | | 7.38 | | 0.01 | |  |  | 15 |
| Antioxidant activity | | 28.40 | | 0.51 | |  |  | 15 |
| pH | | 3.41 | | 0.02 | |  |  | 15 |
| TSS | | 18.83 | | 0.10 | |  |  | 15 |
| **α-terpinen-7-al NC** | |  | |  | |  |  |  |
| % Weight loss | | 2.06 | | 0.01 | | ANOVA and Duncan’s post- hoc test | P<0.05 | 15 |
| Firmness (N) | | 0.40 | | 0.01 | |  |  | 15 |
| Total phenolic content | | 7.35 | | 0.03 | |  |  | 15 |
| Antioxidant activity | | 28.55 | | 0.09 | |  |  | 15 |
| pH | | 3.42 | | 0.02 | |  |  | 15 |
| TSS | | 18.87 | | 0.05 | |  |  | 15 |
| ***C. cyminum* TE** | |  | |  | |  |  |  |
| **200 µg/mL** | |  | |  | |  |  |  |
| % Weight loss | | 2.25 | | 0.01 | | ANOVA and Duncan’s post- hoc test | P<0.05 | 15 |
| Firmness (N) | | 0.46 | | 0.03 | |  |  | 15 |
| Total phenolic content | | 9.52 | | 0.03 | |  |  | 15 |
| Antioxidant activity | | 38.87 | | 0.11 | |  |  | 15 |
| pH | | 3.42 | | 0.01 | |  |  | 15 |
| TSS | | 19.28 | | 0.07 | |  |  | 15 |
| **400 µg/mL** | |  | |  | |  |  |  |
| % Weight loss | | 1.96 | | 0.01 | | ANOVA and Duncan’s post- hoc test | P<0.05 | 15 |
| Firmness (N) | | 0.47 | | 0.02 | |  |  | 15 |
| Total phenolic content | | 9.54 | | 0.02 | |  |  | 15 |
| Antioxidant activity | | 38.32 | | 0.03 | |  |  | 15 |
| pH | | 3.42 | | 0.03 | |  |  | 15 |
| TSS | | 19.24 | | 0.14 | |  |  | 15 |
| **600 µg/mL** | |  | |  | |  |  |  |
| % Weight loss | | 1.84 | | 0.01 | | ANOVA and Duncan’s post- hoc test | P<0.05 | 15 |
| Firmness (N) | | 0.47 | | 0.02 | |  |  | 15 |
| Total phenolic content | | 9.67 | | 0.02 | |  |  | 15 |
| Antioxidant activity | | 39.56 | | 0.31 | |  |  | 15 |
| pH | | 3.42 | | 0.01 | |  |  | 15 |
| TSS | | 18.77 | | 0.08 | |  |  | 15 |
| **800 µg/mL** | |  | |  | |  |  |  |
| % Weight loss | | 1.95 | | 0.01 | | ANOVA and Duncan’s post- hoc test | P<0.05 | 15 |
| Firmness (N) | | 0.48 | | 0.01 | |  |  | 15 |
| Total phenolic content | | 9.87 | | 0.03 | |  |  | 15 |
| Antioxidant activity | | 40.62 | | 0.33 | |  |  | 15 |
| pH | | 3.43 | | 0.03 | |  |  | 15 |
| TSS | | 18.72 | | 0.26 | |  |  | 15 |
| **1,000 µg/mL** | |  | |  | |  |  |  |
| % Weight loss | | 1.77 | | 0.01 | | ANOVA and Duncan’s post- hoc test | P<0.05 | 15 |
| Firmness (N) | | 0.51 | | 0.01 | |  |  | 15 |
| Total phenolic content | | 10.28 | | 0.03 | |  |  | 15 |
| Antioxidant activity | | 43.51 | | 0.26 | |  |  | 15 |
| pH | | 3.43 | | 0.03 | |  |  | 15 |
| TSS | | 18.71 | | 0.04 | |  |  | 15 |
| **Cumin aldehyde TE** | |  | |  | |  |  |  |
| **20 µg/mL** | |  | |  | |  |  |  |
| % Weight loss | | 2.06 | | 0.02 | | ANOVA and Duncan’s post- hoc test | P<0.05 | 15 |
| Firmness (N) | | 0.44 | | 0.01 | |  |  | 15 |
| Total phenolic content | | 8.45 | | 0.16 | |  |  | 15 |
| Antioxidant activity | | 35.28 | | 0.80 | |  |  | 15 |
| pH | | 3.42 | | 0.01 | |  |  | 15 |
| TSS | | 18.29 | | 0.17 | |  |  | 15 |
| **40 µg/mL** | |  | |  | |  |  |  |
| % Weight loss | | 1.81 | | 0.02 | | ANOVA and Duncan’s post- hoc test | P<0.05 | 15 |
| Firmness (N) | | 0.44 | | 0.01 | |  |  | 15 |
| Total phenolic content | | 8.69 | | 0.01 | |  |  | 15 |
| Antioxidant activity | | 36.45 | | 1.79 | |  |  | 15 |
| pH | | 3.42 | | 0.03 | |  |  | 15 |
| TSS | | 18.64 | | 0.50 | |  |  | 15 |
| **60 µg/mL** | |  | |  | |  |  |  |
| % Weight loss | | 1.86 | | 0.01 | | ANOVA and Duncan’s post- hoc test | P<0.05 | 15 |
| Firmness (N) | | 0.45 | | 0.01 | |  |  | 15 |
| Total phenolic content | | 8.38 | | 0.38 | |  |  | 15 |
| Antioxidant activity | | 36.95 | | 1.85 | |  |  | 15 |
| pH | | 3.42 | | 0.01 | |  |  | 15 |
| TSS | | 18.70 | | 0.11 | |  |  | 15 |
| **80 µg/mL** | |  | |  | |  |  |  |
| % Weight loss | | 1.66 | | 0.01 | | ANOVA and Duncan’s post- hoc test | P<0.05 | 15 |
| Firmness (N) | | 0.46 | | 0.01 | |  |  | 15 |
| Total phenolic content | | 9.79 | | 0.01 | |  |  | 15 |
| Antioxidant activity | | 37.11 | | 0.63 | |  |  | 15 |
| pH | | 3.43 | | 0.03 | |  |  | 15 |
| TSS | | 19.40 | | 0.34 | |  |  | 15 |
| **100 µg/mL** | |  | |  | |  |  |  |
| % Weight loss | | 1.53 | | 0.01 | | ANOVA and Duncan’s post- hoc test | P<0.05 | 15 |
| Firmness (N) | | 0.48 | | 0.01 | |  |  | 15 |
| Total phenolic content | | 9.84 | | 0.01 | |  |  | 15 |
| Antioxidant activity | | 37.35 | | 1.48 | |  |  | 15 |
| pH | | 3.43 | | 0.03 | |  |  | 15 |
| TSS | | 19.27 | | 0.39 | |  |  | 15 |
| **α-Terpinen-7-al TE** | |  | |  | |  |  |  |
| **20 µg/mL** | |  | |  | |  |  |  |
| % Weight loss | | 3.34 | | 0.01 | | ANOVA and Duncan’s post- hoc test | P<0.05 | 15 |
| Firmness (N) | | 0.34 | | 0.03 | |  |  | 15 |
| Total phenolic content | | 7.85 | | 0.06 | |  |  | 15 |
| Antioxidant activity | | 32.93 | | 0.21 | |  |  | 15 |
| pH | | 3.33 | | 0.02 | |  |  | 15 |
| TSS | | 19.71 | | 0.20 | |  |  | 15 |
| **40 µg/mL** | |  | |  | |  |  |  |
| % Weight loss | | 3.22 | | 0.01 | | ANOVA and Duncan’s post- hoc test | P<0.05 | 15 |
| Firmness (N) | | 0.35 | | 0.01 | |  |  | 15 |
| Total phenolic content | | 7.76 | | 0.02 | |  |  | 15 |
| Antioxidant activity | | 33.54 | | 0.19 | |  |  | 15 |
| pH | | 3.37 | | 0.01 | |  |  | 15 |
| TSS | | 19.77 | | 0.03 | |  |  | 15 |
| **60 µg/mL** | |  | |  | |  |  |  |
| % Weight loss | | 2.15 | | 0.01 | | ANOVA and Duncan’s post- hoc test | P<0.05 | 15 |
| Firmness (N) | | 0.36 | | 0.01 | |  |  | 15 |
| Total phenolic content | | 8.26 | | 0.03 | |  |  | 15 |
| Antioxidant activity | | 33.88 | | 0.08 | |  |  | 15 |
| pH | | 3.35 | | 0.01 | |  |  | 15 |
| TSS | | 20.12 | | 0.04 | |  |  | 15 |
| **80 µg/mL** | |  | |  | |  |  |  |
| % Weight loss | | 2.30 | | 0.01 | | ANOVA and Duncan’s post- hoc test | P<0.05 | 15 |
| Firmness (N) | | 0.35 | | 0.02 | |  |  | 15 |
| Total phenolic content | | 9.26 | | 0.03 | |  |  | 15 |
| Antioxidant activity | | 34.27 | | 0.25 | |  |  | 15 |
| pH | | 3.37 | | 0.02 | |  |  | 15 |
| TSS | | 20.20 | | 0.10 | |  |  | 15 |
| **100 µg/mL** | |  | |  | |  |  |  |
| % Weight loss | | 2.16 | | 0.01 | | ANOVA and Duncan’s post- hoc test | P<0.05 | 15 |
| Firmness (N) | | 0.38 | | 0.01 | |  |  | 15 |
| Total phenolic content | | 9.55 | | 0.05 | |  |  | 15 |
| Antioxidant activity | | 34.65 | | 0.12 | |  |  | 15 |
| pH | | 3.39 | | 0.01 | |  |  | 15 |
| TSS | | 20.22 | | 0.08 | |  |  | 15 |
| **Table 5**: Quality of grape berries obtained from all treatments after 20 days | | | | | | | |  |
| **CC** | |  | |  | |  | |  |
| % Weight loss | | 5.08 | | 0.13 | | ANOVA and Duncan’s post- hoc test | P<0.05 | 15 |
| Firmness (N) | | 0.21 | | 0.00 | |  |  | 15 |
| Total phenolic content | | 3.30 | | 0.01 | |  |  | 15 |
| Antioxidant activity | | 13.88 | | 0.00 | |  |  | 15 |
| pH | | 3.39 | | 0.01 | |  |  | 15 |
| TSS | | 18.39 | | 0.28 | |  |  | 15 |
| **PC** | |  | |  | |  |  |  |
| % Weight loss | | 8.39 | | 0.22 | | ANOVA and Duncan’s post-hoc test | P<0.05 | 15 |
| Firmness (N) | | 0.13 | | 0.01 | |  |  | 15 |
| Total phenolic content | | 1.89 | | 0.00 | |  |  | 15 |
| Antioxidant activity | | 8.50 | | 0.02 | |  |  | 15 |
| pH | | 3.22 | | 0.01 | |  |  | 15 |
| TSS | | 16.50 | | 0.22 | |  |  | 15 |
| ***C. cyminum* NC** | |  | |  | |  |  |  |
| % Weight loss | | 5.16 | | 0.48 | | ANOVA and Duncan’s post- hoc test | P<0.05 | 15 |
| Firmness (N) | | 0.19 | | 0.01 | |  |  | 15 |
| Total phenolic content | | 3.46 | | 0.01 | |  |  | 15 |
| Antioxidant activity | | 12.91 | | 0.02 | |  |  | 15 |
| pH | | 3.40 | | 0.01 | |  |  | 15 |
| TSS | | 17.58 | | 0.14 | |  |  | 15 |
| **Cumin aldehyde NC** | |  | |  | |  |  |  |
| % Weight loss | | 5.28 | | 0.09 | | ANOVA and Duncan’s post- hoc test | P<0.05 | 15 |
| Firmness (N) | | 0.19 | | 0.02 | |  |  | 15 |
| Total phenolic content | | 3.44 | | 0.01 | |  |  | 15 |
| Antioxidant activity | | 13.38 | | 0.01 | |  |  | 15 |
| pH | | 3.40 | | 0.01 | |  |  | 15 |
| TSS | | 17.75 | | 0.16 | |  |  | 15 |
| **α-terpinen-7-al NC** | |  | |  | |  |  |  |
| % Weight loss | | 5.36 | | 0.08 | | ANOVA and Duncan’s post- hoc test | P<0.05 | 15 |
| Firmness (N) | | 0.17 | | 0.04 | |  |  | 15 |
| Total phenolic content | | 3.42 | | 0.01 | |  |  | 15 |
| Antioxidant activity | | 13.51 | | 0.02 | |  |  | 15 |
| pH | | 3.39 | | 0.01 | |  |  | 15 |
| TSS | | 17.69 | | 0.14 | |  |  | 15 |
| ***C. cyminum* TE** | |  | |  | |  |  |  |
| **200 µg/mL** | |  | |  | |  |  |  |
| % Weight loss | | 5.69 | | 0.26 | | ANOVA and Duncan’s post-hoc test | P<0.05 | 15 |
| Firmness (N) | | 0.21 | | 0.01 | |  |  | 15 |
| Total phenolic content | | 4.41 | | 0.02 | |  |  | 15 |
| Antioxidant activity | | 18.19 | | 0.01 | |  |  | 15 |
| pH | | 3.39 | | 0.02 | |  |  | 15 |
| TSS | | 17.57 | | 0.11 | |  |  | 15 |
| **400 µg/mL** | |  | |  | |  |  |  |
| % Weight loss | | 5.09 | | 0.19 | | ANOVA and Duncan’s post-hoc test | P<0.05 | 15 |
| Firmness (N) | | 0.21 | | 0.01 | |  |  | 15 |
| Total phenolic content | | 4.43 | | 0.02 | |  |  | 15 |
| Antioxidant activity | | 17.90 | | 0.01 | |  |  | 15 |
| pH | | 3.40 | | 0.01 | |  |  | 15 |
| TSS | | 17.60 | | 0.22 | |  |  | 15 |
| **600 µg/mL** | |  | |  | |  |  |  |
| % Weight loss | | 4.76 | | 0.49 | | ANOVA and Duncan’s post- hoc test | P<0.05 | 15 |
| Firmness (N) | | 0.22 | | 0.01 | |  |  | 15 |
| Total phenolic content | | 4.49 | | 0.03 | |  |  | 15 |
| Antioxidant activity | | 18.50 | | 0.01 | |  |  | 15 |
| pH | | 3.40 | | 0.01 | |  |  | 15 |
| TSS | | 17.64 | | 0.21 | |  |  |  |
| **800 µg/mL** | |  | |  | |  |  |  |
| % Weight loss | | 4.91 | | 0.20 | | ANOVA and Duncan’s post-hoc test | P<0.05 | 15 |
| Firmness (N) | | 0.22 | | 0.01 | |  |  | 15 |
| Total phenolic content | | 4.60 | | 0.02 | |  |  | 15 |
| Antioxidant activity | | 18.99 | | 0.01 | |  |  | 15 |
| pH | | 3.42 | | 0.02 | |  |  | 15 |
| TSS | | 17.98 | | 0.24 | |  |  | 15 |
| **1,000 µg/mL** | |  | |  | |  |  |  |
| % Weight loss | | 4.37 | | 0.16 | | ANOVA and Duncan’s post- hoc test | P<0.05 | 15 |
| Firmness (N) | | 0.24 | | 0.01 | |  |  | 15 |
| Total phenolic content | | 4.79 | | 0.01 | |  |  | 15 |
| Antioxidant activity | | 20.46 | | 0.02 | |  |  | 15 |
| pH | | 3.42 | | 0.02 | |  |  | 15 |
| TSS | | 18.06 | | 0.26 | |  |  | 15 |
| **Cumin aldehyde TE** | |  | |  | |  |  |  |
| **20 µg/mL** | |  | |  | |  |  |  |
| % Weight loss | | 5.09 | | 0.11 | | ANOVA and Duncan’s post- hoc test | P<0.05 | 15 |
| Firmness (N) | | 0.20 | | 0.01 | |  |  | 15 |
| Total phenolic content | | 3.94 | | 0.01 | |  |  | 15 |
| Antioxidant activity | | 16.50 | | 0.01 | |  |  | 15 |
| pH | | 3.38 | | 0.01 | |  |  | 15 |
| TSS | | 17.14 | | 0.33 | |  |  | 15 |
| **40 µg/mL** | |  | |  | |  |  |  |
| % Weight loss | | 4.51 | | 0.25 | | ANOVA and Duncan’s post- hoc test | P<0.05 | 15 |
| Firmness (N) | | 0.20 | | 0.02 | |  |  | 15 |
| Total phenolic content | | 4.06 | | 0.02 | |  |  | 15 |
| Antioxidant activity | | 17.08 | | 0.02 | |  |  | 15 |
| pH | | 3.39 | | 0.01 | |  |  | 15 |
| TSS | | 17.43 | | 0.34 | |  |  | 15 |
| **60 µg/mL** | |  | |  | |  |  |  |
| % Weight loss | | 4.68 | | 0.28 | | ANOVA and Duncan’s post- hoc test | P<0.05 | 15 |
| Firmness (N) | | 0.21 | | 0.01 | |  |  | 15 |
| Total phenolic content | | 4.02 | | 0.01 | |  |  | 15 |
| Antioxidant activity | | 17.24 | | 0.02 | |  |  | 15 |
| pH | | 3.39 | | 0.01 | |  |  | 15 |
| TSS | | 17.47 | | 0.33 | |  |  | 15 |
| **80 µg/mL** | |  | |  | |  |  |  |
| % Weight loss | | 4.24 | | 0.39 | | ANOVA and Duncan’s post- hoc test | P<0.05 | 15 |
| Firmness (N) | | 0.21 | | 0.01 | |  |  | 15 |
| Total phenolic content | | 4.57 | | 0.01 | |  |  | 15 |
| Antioxidant activity | | 17.35 | | 0.01 | |  |  | 15 |
| pH | | 3.40 | | 0.01 | |  |  | 15 |
| TSS | | 18.17 | | 0.25 | |  |  | 15 |
| **100 µg/mL** | |  | |  | |  |  |  |
| % Weight loss | | 3.88 | | 0.15 | | ANOVA and Duncan’s post-hoc test | P<0.05 | 15 |
| Firmness (N) | | 0.22 | | 0.01 | |  |  | 15 |
| Total phenolic content | | 4.59 | | 0.02 | |  |  | 15 |
| Antioxidant activity | | 17.70 | | 0.01 | |  |  | 15 |
| pH | | 3.40 | | 0.00 | |  |  | 15 |
| TSS | | 18.22 | | 0.30 | |  |  | 15 |
| **α-Terpinen-7-al TE** | |  | |  | |  |  |  |
| **20 µg/mL** | |  | |  | |  |  |  |
| % Weight loss | | 8.21 | | 0.15 | | ANOVA and Duncan’s post-hoc test | P<0.05 | 15 |
| Firmness (N) | | 0.16 | | 0.02 | |  |  | 15 |
| Total phenolic content | | 3.65 | | 0.02 | |  |  | 15 |
| Antioxidant activity | | 15.44 | | 0.01 | |  |  | 15 |
| pH | | 3.30 | | 0.01 | |  |  | 15 |
| TSS | | 18.40 | | 0.18 | |  |  | 15 |
| **40 µg/mL** | |  | |  | |  |  | 15 |
| % Weight loss | | 8.01 | | 0.44 | | ANOVA and Duncan’s post- hoc test | P<0.05 | 15 |
| Firmness (N) | | 0.17 | | 0.01 | |  |  | 15 |
| Total phenolic content | | 3.62 | | 0.02 | |  |  | 15 |
| Antioxidant activity | | 15.65 | | 0.01 | |  |  | 15 |
| pH | | 3.35 | | 0.01 | |  |  | 15 |
| TSS | | 18.47 | | 0.21 | |  |  | 15 |
| **60 µg/mL** | |  | |  | |  |  |  |
| % Weight loss | | 5.84 | | 0.29 | | ANOVA and Duncan’s post- hoc test | P<0.05 | 15 |
| Firmness (N) | | 0.17 | | 0.02 | |  |  | 15 |
| Total phenolic content | | 3.84 | | 0.04 | |  |  | 15 |
| Antioxidant activity | | 15.82 | | 0.02 | |  |  | 15 |
| pH | | 3.34 | | 0.01 | |  |  | 15 |
| TSS | | 18.83 | | 0.18 | |  |  | 15 |
| **80 µg/mL** | |  | |  | |  |  |  |
| % Weight loss | | 5.65 | | 0.36 | | ANOVA and Duncan’s post- hoc test | P<0.05 | 15 |
| Firmness (N) | | 0.18 | | 0.02 | |  |  | 15 |
| Total phenolic content | | 4.35 | | 0.02 | |  |  | 15 |
| Antioxidant activity | | 16.06 | | 0.02 | |  |  | 15 |
| pH | | 3.35 | | 0.01 | |  |  | 15 |
| TSS | | 18.90 | | 0.22 | |  |  | 15 |
| **100 µg/mL** | |  | |  | |  |  |  |
| % Weight loss | | 5.42 | | 0.47 | | ANOVA and Duncan’s post- hoc test | P<0.05 | 15 |
| Firmness (N) | | 0.18 | | 0.03 | |  |  | 15 |
| Total phenolic content | | 4.47 | | 0.02 | |  |  | 15 |
| Antioxidant activity | | 16.17 | | 0.02 | |  |  | 15 |
| pH | | 3.36 | | 0.01 | |  |  | 15 |
| TSS | | 20.22 | | 0.08 | |  |  | 15 |
